# Study on the Separation, Identification, and Quality Control Methods of Oligosaccharide Components in Wine-Processed Polygonati Rhizoma

**DOI:** 10.3390/foods15030584

**Published:** 2026-02-05

**Authors:** Hong Guo, Haonan Wu, Rui Yao, Zhe Li, Xianlong Cheng, Yongqiang Lin, Feng Wei, Yazhong Zhang, Jingzhe Pu, Jianbo Yang, Ying Wang, Jia Chen, Wenguang Jing, Xiaohan Guo

**Affiliations:** 1 National Institutes for Food and Drug Control, Beijing 102629, China; 2 School of Functional Food and Wine, Shenyang Pharmaceutical University, Shenyang 110016, China; 3 Anhui Institute for Food and Drug Control, Hefei 230051, China

**Keywords:** Polygonati Rhizoma, nine cycles of steaming and drying, difructose anhydrides, extraction and isolation, structural identification, content

## Abstract

This study aimed to identify oligosaccharide quality markers in wine-processed Polygonati Rhizoma (WPR) and to examine how sugar components change during its repeated steaming and drying, correlating these changes with the color of the processed slices. Seven oligosaccharides were extracted from WPR using water extraction, ethanol precipitation, and preparative liquid chromatography. Identified by NMR with purities above 93%, they were DFA III (**1**), DFA VII (**2**), DFA II (**3**), *β*-D-Fructo*furanose*-1,2′:2,1′-*β*-D-Fructo*furanose* (**4**), DFA I (**5**), sucrose (**6**), and 1-kestose (**7**). Compounds **1**–**5** were newly isolated from WPR, and their contents increased post-processing. Measurements of color values, total reducing sugars, and the abundant DFA III and DFA I at different processing stages revealed a progressive darkening of the slices with more steaming cycles, showing a strong correlation between total color difference ($E_{\mathrm{ab}}^{*}$) and sugar component changes. Additionally, DFA III and DFA I levels were much higher in commercial WPR than in raw material, suggesting these difructose anhydrides as potential markers for WPR.

## 1. Introduction

Polygonati Rhizoma (PR), commonly known as Huangjing, is a perennial herb of the *Polygonatum* genus in the Liliaceae family, primarily distributed in East and Southeast Asia. In its raw form, it has a faint odor, a sweet taste, and a sticky texture when chewed. In traditional Chinese medicine theory, it is associated with the spleen, lung, and kidney meridians. It is attributed with functions such as replenishing qi, nourishing yin, strengthening the spleen, moistening the lung, and tonifying the kidney [1], where “qi” refers to the vital energy or functional capacity of the body, and “yin” represents the moistening, nourishing, and material substrates of the body. This dual action is believed to alleviate fatigue (qi deficiency) and dryness (yin deficiency). Historically, it has been used for conditions including spleen-stomach qi deficiency, fatigue, and internal heat with thirst. The 2025 edition of the *Pharmacopoeia of the People’s Republic of China* includes three botanical origins: *Polygonatum kingianum* Coll. et Hemsl., *Polygonatum sibiricum* Red., and *Polygonatum cyrtonema* Hua [1]. As a traditional medicinal and edible resource, PR is widely used in the functional food and dietary supplement industries, with over 200 health products developed to date [2]. It holds significant health value. However, the raw material contains irritant components that cause a numbing sensation on the tongue. Processing is required to eliminate this irritancy and enhance its tonic effects [3].

Current research on the chemical constituents of PR has largely focused on polysaccharides and saponins [4]. However, polysaccharide content tends to decrease during processing [5]. Notably, the 2025 Pharmacopoeia removed the content determination item for wine-steamed PR, specifying only that it must be “stewed or steamed thoroughly,” reflecting the current lack of comprehensive quality control markers. It is worth noting that oligosaccharides, although abundant in PR, have been relatively understudied. Our research group previously established an analytical method for PR oligosaccharides [6] and observed a significant increase in the mass spectrometric response of certain saccharides during the processing of wine-steamed PR, suggesting their potential as processing markers.

In traditional empirical identification, the processing of PR emphasizes “assessing quality based on form,” with records stating that “darker color indicates better quality.” The *Bencao Tujing* further describes high-quality processed PR as “shiny black as lacquer, sweet as malt sugar.” Modern research also indicates that saccharides are one of the major active components in PR, possessing various pharmacological effects such as anti-inflammatory [7], anti-aging [8], improvement of osteoporosis [9], and hypoglycemic activity [10]. Among them, difructose anhydride III (DFA III) and related oligosaccharides have been found to exhibit properties such as low caloric value and prebiotic activity [11], along with reported effects on mineral absorption improvement and immunomodulation [12]. With half the sweetness but only 1/15 the caloric content of sucrose, these components show promise as potential indicators for evaluating processing quality.

Therefore, to systematically characterize the oligosaccharides that increase significantly during the processing of wine-steamed PR and to explore the correlation between the traditional criterion of “shiny black as lacquer” and modern chemical indicators, this study used wine-steamed PR as the research material. Techniques including water extraction followed by alcohol precipitation, membrane filtration, gel column chromatography, and preparative liquid chromatography were employed to isolate and purify the oligosaccharide fractions. Structural identification was performed using high-resolution mass spectrometry and nuclear magnetic resonance spectroscopy. Simultaneously, colorimetric analysis and saccharide content determination were combined to systematically investigate the correlation between the color of the herbal slices and the dynamic changes in saccharide components during the nine cycles of steaming and drying. This work aims to provide a scientific basis for establishing a quality evaluation system for wine-steamed PR that integrates traditional experience with modern quality control.

## 2. Materials and Methods

### 2.1. Materials

Instruments: The equipment involved in oligosaccharide extraction includes a constant temperature water bath (Tianjin Test Instrument Co., Ltd., Tianjin, China), a rotary evaporator (IKA RA10), a high-speed refrigerated centrifuge (Shanghai Techcomp Laboratory Instrument Co., Ltd., Shanghai, China), and a vacuum freeze dryer (Beijing Songyuan Huaxing Technology Development Co., Ltd., Beijing, China). Subsequently, spiral-wound ultrafiltration membranes and nanofiltration membranes (PES, Shandong Bona Technology Group, Jinan, China) were used for preliminary enrichment of the oligosaccharides. Purification to obtain monomers was achieved through Sephadex G15 gel (Beijing Solarbio Science & Technology Co., Ltd., Beijing, China) and a Shimadzu RID-10A preparative high-performance liquid chromatography system (Shimadzu Corporation, Japan, Kyoto, Japan), using an XBridge BEH Amide OBD Prep Column (19 × 250 mm, 5 µm, Waters Corporation, Milford, MA, USA) as the preparative column. The structure of the oligosaccharides was determined using an Orbitrap Fusion Lumos high-resolution mass spectrometer (Thermo Fisher Scientific, USA, Waltham, MA, USA) and an AVANCE NEO 600M nuclear magnetic resonance spectrometer (Bruker Corporation, Billerica, MA, USA). The spectrophotometer was produced by 3 nh, model YS6060. The electronic balance used in the experiments was from Sartorius Scientific Instruments Co., Ltd., Beijing, China (with a precision of 1/100,000). Extraction was performed using an ultrasonic cleaner (Kunshan Ultrasonic Instruments Co., Ltd., Kunshan, China), and content determination was carried out with a Vanquish Flex charged aerosol detector (CAD) ultra-high-performance liquid chromatography system (Thermo Fisher Scientific, Waltham, MA, USA). The analytical columns used included an Ecosil Grace Carbohydrate ES, a Shodex HILICpak VG-50 4E, and an XBridge BEH Amide Column (4.6 × 250 mm, 5 µm).

Reagents: The solvent used for NMR was deuterium oxide (Cambridge Isotope Laboratories, Inc., Tewksbury, MA, USA), with sodium 3-(trimethylsilyl)propionate-2,2,3,3-d4 (Alfa Aesar, Waltham, MA, USA) as the internal standard. Conventional reagents were of analytical grade, reagents for HPLC were of chromatographic grade, and all experimental solutions were prepared using ultrapure water produced by a water purification system (Millipore, Merck KGaA, Darmstadt, Germany). The yellow rice wine used for processing *Polygonatum kingianum* was sourced from Zhejiang Deqing Moganshan Wine Industry Co., Ltd. (Huzhou, China), with an alcohol content of 10.0% vol, batch number 20241228.

Tested Materials: WPR sample J1 (batch No. TRT202301) was purchased from Beijing Tongrentang Group (Beijing, China); raw PK material K0 (batch No. YNDH202401) was collected in Dehong, China; raw PC material C0 (batch No. AHBZ202401) was collected in Bozhou, China; raw PS material S0 (batch No. HNLY202401) was collected in Luoyang, China. All materials were identified by Professor Jianbo Yang (National institutes for food and drug control) and Jingzhe Pu (Anhui institute for food and drug control) as the dried rhizomes of *Polygonatum kingianum* Coll. et Hemsl., *Polygonatum cyrtonema* Hua, and *Polygonatum sibiricum* Red., respectively. WPR samples processed through nine cycles of steaming and drying were prepared in the laboratory and preserved at the National Institutes for Food and Drug Control. The preparation method was as follows: Raw PK material (designated K0) was mixed with yellow rice wine in a ratio of 5:1 (by weight), moistened until the wine was fully absorbed and the material softened. It was then placed in a steamer with an appropriate amount of water and steamed for 3 h, sliced, and air-dried for 48 h to obtain the once-steamed and once-dried sample K1. A portion was retained after each cycle. The remaining slices were mixed with the residual liquid, and the steaming process was repeated with added water. This procedure was carried out nine times to obtain samples K1 through K9. Samples of PC (C0–C9) and PS (S0–S9) were prepared following the same procedure. Information for all laboratory-prepared samples and commercially collected samples is summarized in Table 1, among which samples 42–46 were collected from the market.

The reference standard for 1-kestose (batch No. 2365629, purity 97.5%) was purchased from ANPEL Laboratory Technologies Inc. (Shanghai, China). Reference standards for fructose (batch No. 100231-202309, purity 99.9%), glucose (batch No. 110833-202109, purity 99.8%), and sucrose (batch No. 111507-202406, purity 100%) were obtained from the National Institutes for Food and Drug Control, Beijing, China. Compounds **1** (DFA Ⅲ) and **5** (DFAⅠ) were isolated in the laboratory.

### 2.2. Extraction and Isolation of Oligosaccharides

**Figure 1:**
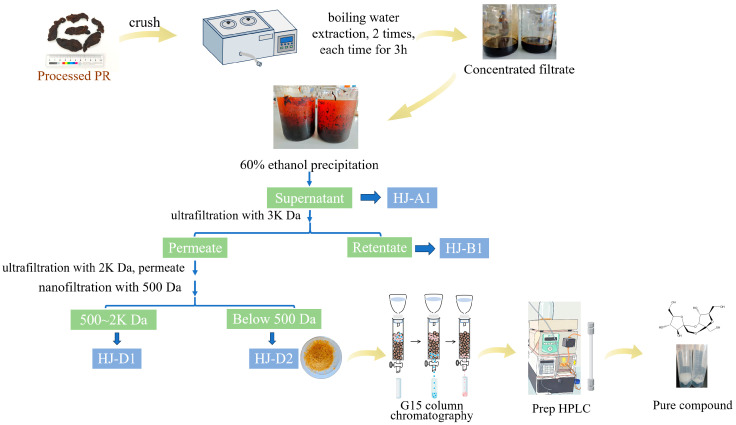
Flowchart of the extraction and isolation process from wine-processed Polygonati Rhizoma.

Referring to a previously established method [6], 1 kg of WPR sample J1 (batch No. TRT202301) was dried, pulverized, and extracted twice with 10 volumes of purified water at 100 °C for 3 h per extraction with intermittent stirring. After filtration, the combined extract was concentrated under reduced pressure to approximately 1 L. To this concentrate, 1.5 L of anhydrous ethanol was added to achieve a final ethanol concentration of 60%. The mixture was left to stand overnight (12 h) at 4 °C. The solution after ethanol precipitation was centrifuged at 9000× *g* for 15 min. The precipitate was discarded, and the supernatants were combined, concentrated under reduced pressure, and pooled to obtain a crude oligosaccharide extract from WPR, designated as HJ-A1 (Figure 1).

A tangential flow filtration system was used to enrich and preliminarily fractionate the oligosaccharides. The total oligosaccharide sample HJ-A1 obtained above was diluted with purified water to about 5 L and filtered through a 0.2 μm membrane to remove insoluble solid materials. The resulting clear solution was sequentially passed through roll-type ultrafiltration and nanofiltration membranes with molecular weight cut-offs (MWCOs) of 3000 Da, 2000 Da, and 500 Da, respectively. Finally, the solution was concentrated using a reverse osmosis membrane. The 3000 Da membrane removed high-molecular-weight polysaccharides and proteins, while the sequential separation with 2000 Da and 500 Da membranes aimed to collect oligosaccharides, with the <500 Da fraction enriching the target disaccharide components. A total of four fractions were obtained: HJ-A1 (aqueous extract after ethanol precipitation), HJ-B1 (components >3000 Da), HJ-D1 (components between 500 Da and 2000 Da), and HJ-D2 (components <500 Da). Each fraction was freeze-dried, yielding the following percentages: HJ-A1: 26.34%, HJ-B1: 8.62%, HJ-D1: 4.49%, and HJ-D2: 22.46%. The HJ-D2 was dissolved in an appropriate amount of water and eluted through a Sephadex G15 gel column (3 × 180 cm) with purified water at a flow rate of 0.3 mL/min. The eluate was collected in glass bottles, 15 mL per bottle, yielding 24 bottles per column. After analysis by HPLC-CAD and subsequent pooling, fractions 2–9 (from the second column, bottle 9) and 1–16 were further separated and purified by preparative liquid chromatography. The mobile phase consisted of acetonitrile (87%)–water (13%), and separation was monitored using a differential refractive index detector (RID) at a flow rate of 7 mL/min. The mobile phase consisted of acetonitrile (87%) and water (13%). The separation was monitored using a differential refractive index detector (RID) at a flow rate of 7 mL/min. Monomeric compounds were obtained by purifying the enriched fractions. The overall extraction and isolation procedure is illustrated in Figure 1

### 2.3. Methods for Structural Identification

Liquid Chromatography: All compounds purified by preparative liquid chromatography were analyzed using HPLC-CAD. The mobile phase consisted of acetonitrile (Solvent A) and water (Solvent B), employing an isocratic elution with 13% B. Following peak integration, the purity of each compound was determined by calculating the ratio of the main peak area to the total peak area.

High-Resolution Mass Spectrometry: The molecular weights of the purified oligosaccharides were determined using an Orbitrap Fusion Lumos high-resolution mass spectrometer. Chromatographic separation was achieved on an XBridge BEH Amide Column (4.6 mm × 250 mm, 5 µm). Detection was performed in negative ion mode. The collision gas was high-purity argon, with an ionization voltage of 3000 V. The sheath gas and auxiliary gas flow rates were set at 45 L/h and 15 L/h, respectively. The instrument was operated with a first-stage resolution of 120,000 and a second-stage resolution of 30,000. The ion transfer tube temperature was maintained at 350 °C, and the vaporizer temperature at 320 °C. The mass scan range for both MS1 and MS2 was set to *m*/*z* 150–1000. Fragmentation was achieved using the Higher-energy Collisional Dissociation mode with a collision energy of 30 eV.

Nuclear Magnetic Resonance Spectroscopy: Approximately 10 mg of the purified oligosaccharide sample was dissolved in 0.6 mL of D_2_O. Using TSP (3-(Trimethylsilyl)-2,2,3,3-tetradeuteropropionic acid sodium salt) as an internal standard, ^1^H NMR, ^13^C NMR, HSQC, HMBC, and ^1^H-^1^H COSY spectra were acquired at 600 MHz on a nuclear magnetic resonance spectrometer.

### 2.4. Quantitative Analysis of Difructose Anhydrides

#### 2.4.1. Chromatographic Conditions for Difructose Anhydrides

Analysis was performed using a CAD. Separation was achieved on an XBridge BEH Amide Column (4.6 mm × 250 mm, 5 µm). The mobile phase consisted of acetonitrile (solvent A) and 0.1% ammonia solution (solvent B), with isocratic elution at 87% A and 13% B. The flow rate was 1.0 mL/min, and the column temperature was maintained at 30 °C. The injection volume was 10 µL. For the CAD, the data acquisition frequency was set at 5 Hz with a filter constant of 2.0 s, and the evaporation temperature was 60 °C.

#### 2.4.2. Sample Preparation for Analysis

Approximately 0.5 g of finely powdered samples (K0–K9, C0–C9, S0–S9, passed through a No. 5 sieve) was accurately weighed and transferred into a stoppered conical flask. A precise volume of 40 mL of 80% ethanol was added. The flask was weighed, subjected to ultrasonication for 30 min, then removed and allowed to cool to room temperature. The weight loss was compensated by adding 80% ethanol. The mixture was shaken well and filtered through a 0.22 µm membrane filter. The subsequent filtrate was collected as the test solution.

#### 2.4.3. Reference Standard Solution Preparation

Reference compounds **1** and **5** were accurately weighed, dissolved, and mixed in 80% ethanol. The solutions were then transferred and made up to volume in 10 mL volumetric flasks, yielding reference standard solutions with concentrations of 0.518 mg/mL and 0.522 mg/mL, respectively. A negative control solution, prepared in the same manner but without the addition of reference standards, was also prepared.

### 2.5. Colorimetric Determination

After the colorimeter was powered on and stabilized, black and white calibration was performed using the instrument’s accessories. The color of PR powder was characterized using the three parameters L*, a*, and b*. The total color difference $E_{\mathrm{ab}}^{*}$ and the degree of color difference between the wine-steamed product and the raw material, Δ$E_{\mathrm{ab}}^{*}$, were calculated as $E_{\mathrm{ab}}^{*}\;=\sqrt{L_{*}^{2}\;+{\;a}_{*}^{2}\;+\;b_{*}^{2}}$. The aperture diameter was 15 mm.

The colorimeter was placed in a stable indoor lighting environment. After powering on and allowing the instrument to stabilize, black-and-white calibration was performed using the provided accessories. The color of PR powder was characterized by three parameters: L*, a*, and b*. Each sample was measured three times, and the total color difference $E_{\mathrm{ab}}^{*}\;=\sqrt{L_{*}^{2}\;+\;a_{*}^{2}\;+\;b_{*}^{2}}$ was calculated, along with Δ$E_{\mathrm{ab}}^{*}$ representing the degree of color difference between wine-steamed Polygonatum and raw materials. The aperture diameter was set to 15 mm. Additionally, visual color changes in WPR powder at different processing stages were photographed under uniform lighting.

### 2.6. Determination of Reducing Sugar Content

According to the Ministry of Agriculture standard NY/T 2742-2015, the total reducing sugar content was determined using the 3,5-dinitrosalicylic acid (DNS) colorimetric method [13]. The DNS reagent was prepared as follows: First, 6.3 g of 3,5-dinitrosalicylic acid was dissolved in 262 mL of 2 mol/L sodium hydroxide solution. This mixture was then added to 500 mL of hot water containing 185 g of potassium sodium tartrate tetrahydrate. Subsequently, 5 g of phenol and 5 g of sodium sulfite were added with continuous stirring until complete dissolution. After cooling, the solution was diluted to 1000 mL with distilled water and stored in a brown bottle for later use.

#### 2.6.1. Preparation of Glucose Standard Curve

A reference stock solution with a concentration of 1 mg/mL was prepared by accurately weighing and dissolving glucose. Aliquots of 0, 0.2, 0.3, 0.4, 0.5, 0.6, 0.8, and 1.0 mL were precisely pipetted into eight test tubes, respectively. The volume in each tube was adjusted to 4.0 mL with water. This was followed by the addition of 5 mL of DNS reagent to each tube. After thorough mixing, the tubes were placed in a boiling water bath for 5 min. The reaction was immediately terminated by cooling under running water, and the volume in each tube was finally made up to 10 mL with water. Using the blank tube (0 mL) to zero the instrument, the absorbance of the series of solutions was measured at 540 nm using ultraviolet–visible(UV) spectrophotometry. A standard curve was established by performing linear regression analysis on the measured absorbance values against the corresponding mass of glucose.

#### 2.6.2. Preparation of Test Solution for Total Reducing Sugars

Approximately 0.1 g of fine powder of PR was accurately weighed into a conical flask. Then, 30 mL of 80% ethanol was added, and the mixture was extracted under reflux in a water bath for 1 h. After cooling, the extract was filtered. The filtrate was transferred to a 50 mL volumetric flask. The residue was washed with 80% ethanol, and the washings were combined into the same volumetric flask. Finally, the volume was made up to the mark with 80% ethanol to obtain the test solution for reducing sugar analysis.

### 2.7. Determination of Total Polysaccharide and Total Oligosaccharide Contents

#### 2.7.1. Preparation of Reference Solution

Anhydrous glucose reference substance (dried to constant weight) weighing 33 mg was precisely weighed and transferred into a 100 mL volumetric flask. After dissolving and diluting with water to the mark, the solution was mixed thoroughly to obtain an anhydrous glucose reference solution with a concentration of 0.33 mg/mL.

#### 2.7.2. Preparation of Standard Curve

Precisely 0, 0.1 mL, 0.2 mL, 0.3 mL, 0.4 mL, 0.5 mL, and 0.6 mL of the reference solution were, respectively, transferred into 10 mL stoppered graduated test tubes. Water or 80% ethanol was added to each tube to reach 2.0 mL, followed by thorough mixing. In an ice-water bath, 0.2% anthrone–sulfuric acid solution was slowly added dropwise to the mark. After mixing and cooling, the tubes were placed in a water bath and maintained for 10 min. They were then immediately transferred to an ice-water bath and cooled for another 10 min. Using tube 1 as the blank, the absorbance of each solution was measured at 582 nm according to the ultraviolet–visible spectrophotometry method. A standard curve was plotted with absorbance as the ordinate and concentration as the abscissa.

#### 2.7.3. Preparation of Test Solutions for Total Polysaccharides and Oligosaccharides

Powdered PR samples (K0–K9, C0–C9, S0–S9, dried to constant weight) weighing 0.25 g were precisely weighed and refluxed with 150 mL of 80% ethanol for 1 h. The mixture was filtered while hot, and the residue was washed three times with 10 mL of hot 80% ethanol each time. After cooling, the filtrate was diluted to volume in a 250 mL volumetric flask. Then, 8 mL of this solution was transferred into a 50 mL volumetric flask and diluted to the mark to obtain the test solution of PR oligosaccharides.

The residue and filter paper were placed in a conical flask and extracted in a boiling water bath for 1 h. After hot filtration, the residue and flask were washed four times with 10 mL of hot water each time. The filtrate and washings were combined, cooled, and diluted to volume in a 250 mL volumetric flask to obtain the test solution of PR polysaccharides.

### 2.8. Analytical Method for Free Sugar Content

#### 2.8.1. Liquid Chromatography Method

The contents of four free sugars—fructose, glucose, sucrose, and kestose—in raw and wine-processed PR were determined using a CAD. Separation was performed on an Ecosil Grace Carbohydrate ES column (5 µm, 250 × 4.6 mm). The mobile phase consisted of acetonitrile (solvent A) and water (solvent B). A gradient elution program was used: 0–18 min, 16–22% B; 18–22 min, 22% B; 28–30 min, 22–30% B; 30–35 min, 30% B; 35–40 min, 30–35% B; 41–47 min, 80% B; 48–55 min, 16% B. The flow rate was 1.0 mL/min, the column temperature was maintained at 30 °C, and the injection volume was 10 µL.

#### 2.8.2. Preparation of Test Solutions

Approximately 0.5 g of fine powder of raw or wine-processed PK was accurately weighed into a stoppered conical flask. A precise volume of 50 mL of 80% ethanol was added. The flask was weighed, subjected to ultrasonication for 30 min, then removed and allowed to cool. The weight loss was compensated by adding 80% ethanol. After shaking well, the mixture was filtered through a 0.22 µm membrane filter. The subsequent filtrate was collected as the test solution.

#### 2.8.3. Preparation of Reference Standard Stock Solutions

The four reference standards were accurately weighed and dissolved to prepare a mixed standard solution with concentrations of 2 mg/mL for fructose, 0.5 mg/mL for glucose, 0.5 mg/mL for sucrose, and 0.25 mg/mL for 1-kestose. A negative control solution, prepared in the same manner but without the addition of fructose, glucose, sucrose, or 1-kestose, was also prepared.

### 2.9. Statistical Analysis

Colorimetric parameters (L*, a*, b*, and Δ$E_{\mathrm{ab}}^{*}$) and sugar content data for the PR samples were imported into the SIMCA 14.1 statistical software. Unsupervised principal component analysis (PCA) was performed. Furthermore, Pearson correlation analysis was conducted between the colorimetric values and the sugar content data.

## 3. Results

To systematically elucidate the key chemical transformations and phenotypic evolution of WPR during the nine-steaming and nine-drying process, the structures of the isolated oligosaccharides were identified. Quantitative monitoring was conducted on indicators reflecting the dynamic changes in carbohydrate components, including reducing sugars and total polysaccharides as key substrates and products, as well as characteristic DFAs (DFA Ⅲ and DFA Ⅰ). Simultaneously, colorimetric values representing the appearance quality of the processed slices were measured, and the intrinsic correlations between these chemical indicators and physical characteristics were thoroughly investigated.

### 3.1. Results of Structural Identification

After separation by preparative liquid chromatography, compounds **1** (*t*_R_ = 34.46 min, 38.42 mg), **2** (*t*_R_ = 36.54 min, 40.65 mg), **3** (*t*_R_ = 40.22 min, 30.78 mg), **4** (*t*_R_ = 43.57 min, 27.34 mg), **5** (*t*_R_ = 46.45 min, 41.26 mg), **6** (*t*_R_ = 64.23 min, 31.78 mg), and **7** (*t*_R_ = 89.04 min, 17.02 mg) were obtained.

**Figure 2:**
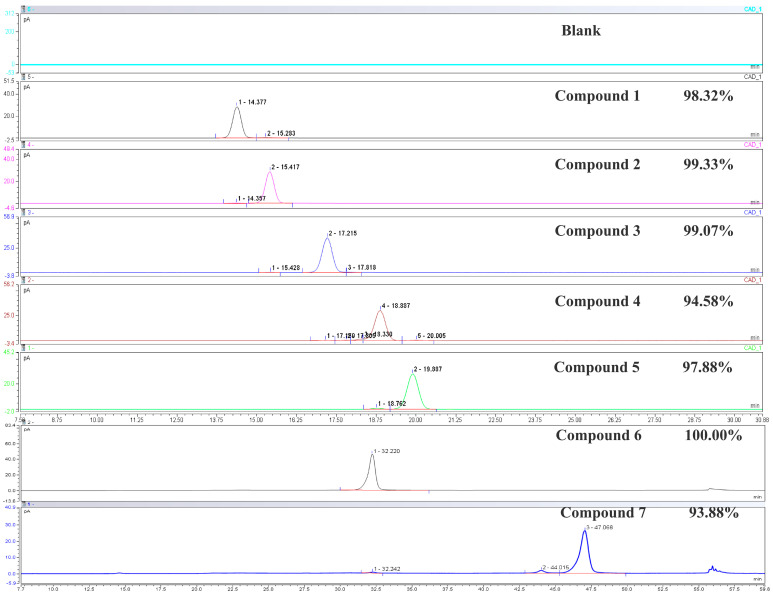
HPLC chromatogram of sugar disaccharides from wine-processed Polygonati Rhizoma.

The analytical results of the compounds are shown in Figure 2. HPLC analysis revealed that, except for compounds **4** and **7**, all compounds exhibited purities above 97%. The purities of compounds **4** and **7** were above 93%, meeting the purity requirements for NMR analysis. The NMR data are presented in Table S1. The ^1^H NMR and ^13^C NMR spectra are shown in Figure S1.

Compound **1**: White amorphous solid. HR-ESI-MS displayed a quasi-molecular ion peak at *m*/*z*: 323.09772 [M–H]^−^ (C_12_H_19_O_10_^−^, calculated 323.09727, error 1.383 ppm). ^1^H-NMR (600 MHz, D_2_O) *δ*: 4.06 (d, *J* = 13.2 Hz, H-1a), 3.77 (d, *J* = 13.2 Hz, H-1b), 4.16 (d, *J* = 4.2 Hz, H-3), 3.89 (dd, *J* = 1.2, 4.2 Hz, H-4), 4.11 (m, *J* = 1.2, 3.0, 3.6 Hz, H-5), 3.90 (dd, *J* = 3.6, 12.6 Hz, H-6a), 3.82 (dd, *J* = 3.0, 12.6 Hz, H-6b), 3.71 (d, *J* = 12 Hz, H-1′a), 3.65 (d, *J* = 12 Hz, H-1′b), 4.34 (d, *J* = 7.0 Hz, H-3′), 4.67 (dd, *J* = 1.2, 7.0 Hz, H-4′), 3.71 (m, *J* = 1.2, 4.8, 5.4 Hz, H-5′), 3.76 (dd, *J* = 4.8, 12.6 Hz, H-6′a), 3.65 (dd, *J* = 5.4, 12.6 Hz, H-6′b). ^13^C-NMR (150 MHz, D_2_O) δ: 61.81 (C-1), 106.43 (C-2), 84.01 (C-3), 75.30 (C-4), 84.71 (C-5), 63.92 (C-6), 66.27 (C-1′), 104.19 (C-2′), 81.86 (C-3′), 72.19 (C-4′), 82.97 (C-5′), 63.43 (C-6′). Comparison of the above data with those reported in the literature [14] showed they were in good agreement, confirming Compound **1** as *α*-D-Fructo*furanose*-1,2′:2,3′-*β*-D-Fructo*furanose* (DFA Ⅲ).

Compound **2**: Pale yellow amorphous solid. ESI-MS *m*/*z*: 323.09775 [M–H]^−^ (C_12_H_19_O_10_^−^, calculated 323.09727, error 1.476 ppm), suggesting a symmetrical structure. ^1^H-NMR (600 MHz, D_2_O) δ: 4.02 (d, *J* = 12.6 Hz, H-1a, H-1′a), 3.84 (d, *J* = 12.6 Hz, H-1b, H-1′b), 4.06 (d, *J* = 3.6 Hz, H-3, H-3′), 3.97 (dd, *J* = 3.6, 6.0 Hz, H-4, H-4′), 4.02 (m, *J* = 3.0, 6.0 Hz, H-5, H-5′), 3.82 (dd, *J* = 3.0, 12.6 Hz, H-6a, H-6′a), 3.70 (dd, *J* = 6.0, 12.6 Hz, H-6b, H-6′b). ^13^C-NMR (150 MHz, D_2_O) δ: 63.94 (C-1, C-1′), 106.65 (C-2, C-2′), 82.81 (C-3, C-3′), 79.63 (C-4, C-4′), 85.80 (C-5, C-5′), 63.84 (C-6, C-6′), comparison of the above data with those reported in the literature [14] indicated a high degree of consistency, confirming Compound **2** as *α*-D- Fructo*furanose*-1,2′:2,1′-*α*-D- Fructo*furanose* (DFA Ⅶ).

Compound **3**: White amorphous solid. ESI-MS *m*/*z*: 323.09775 [M–H]^−^ (C_12_H_19_O_10_^−^, calculated 323.09727, error 1.476 ppm). ^1^H-NMR (600 MHz, D_2_O) *δ*: 4.04 (d, *J* = 12.0 Hz, H-1a), 3.76 (d, *J* = 12.0 Hz), H-1b), 4.20 (d, *J* = 1.2 Hz), H-3), 4.10 (dd, *J* = 1.2, 2.4 Hz, H-4), 3.98 (m, *J* = 2.4, 5.4, 6.0 Hz, H-5), 3.76 (dd, *J* = 6.0,12.6 Hz, H-6a), 3.75 (dd, *J* = 5.4, 12.6 Hz, H-6b), 3.71 (d, *J* = 12.6 Hz, H-1′a), 3.67 (d, *J* = 12.6 Hz, H-1′b), 3.88 (d, *J* = 7.8 Hz, H-3′), 4.14 (dd, *J* = 7.2, 7.8 Hz, H-4′), 3.91 (m, *J* = 3.6, 6.6, 7.2 Hz, H-5′), 3.80 (dd, *J* = 3.6, 12.6 Hz, H-6′a), 3.68 (dd, *J* = 6.6, 12.6 Hz, H-6′b). ^13^C-NMR (150 MHz, D_2_O) δ: 66.23 (C-1), 106.53 (C-2), 76.16 (C-3), 78.78 (C-4), 87.51 (C-5), 65.02 (C-6), 65.07 (C-1′), 100.76 (C-2′), 79.90 (C-3′), 77.03 (C-4′), 83.99 (C-5′), 65.43 (C-6′), comparison of the above data with those reported in the literature [15] showed they were in good agreement, confirming Compound **3** as *β*-D-fructo*furanose*-1,2′:2,3′-*β*-D-fructo*furanose* (DFA Ⅱ).

Compound **4**: White amorphous solid. ESI-MS *m*/*z*: 323.09787 [M–H]^−^ (C_12_H_19_O_10_^−^, calculated 323.09727, error 1.847 ppm), suggesting a symmetrical structure. ^1^H-NMR (600 MHz, D_2_O) δ: 4.03 (d, *J* = 13.2 Hz, H-1a, H-1′a), 3.96 (d, *J* = 12.6 Hz, H-1b, H-1′b), 4.02 (d, *J* = 7.8 Hz, H-3, H-3′), 4.10 (dd, *J* = 1.2, 7.8 Hz, H-4, H-4′), 3.89 (m, *J* = 1.2, 3.6, 6.6 Hz, H-5, H-5′), 3.78 (dd, *J* = 3.6, 12.6 Hz, H-6a, H-6′a), 3.66 (dd, *J* = 6.6, 12.6 Hz, H-6b, H-6′b). ^13^C-NMR (150 MHz, D_2_O) δ: 66.40 (C-1, C-1′), 103.49 (C-2, C-2′), 81.96 (C-3, C-3′), 77.42 (C-4, C-4′), 84.25 (C-5, C-5′), 65.20 (C-6, C-6′), comparison of the above data with those reported in the literature [14] showed they were in good agreement, confirming Compound **4** as *β*-D-Fructo*furanose*-1,2′:2,1′-*β*-D-Fructo*furanose*.

Compound **5**: White amorphous solid. ESI-MS *m*/*z*: 323.09784 [M–H]^−^ (C_12_H_19_O_10_^−^, calculated 323.09727, error 1.754 ppm). ^1^H-NMR (600 MHz, D_2_O) *δ*: 4.16 (d, *J* = 12.0 Hz, H-1a), 3.70 (d, *J* = 12.0 Hz, H-1b), 4.05 (d, *J* = 2.4 Hz, H-3), 3.96 (dd, *J* = 2.4, 5.4 Hz, H-4), 4.03 (m, *J* = 3.0, 5.4 Hz, H-5), 3.87 (dd, *J* = 3.0, 12.6 Hz, H-6a), 3.75 (dd, *J* = 5.4, 12.6 Hz, H-6b), 4.23 (d, *J* = 12.0 Hz, H-1′a), 3.62 (d, *J* = 12.0 Hz, H-1′b), 3.88 (d, *J* = 8.4 Hz, H-3′), 4.13 (dd, *J* = 7.2, 8.4 Hz, H-4′), 3.94 (m, *J* = 3.0, 6.6, 7.2 Hz, H-5′), 3.80 (dd, *J* = 3.0, 12.6 Hz, H-6′a), 3.62 (dd, *J* = 6.6, 12.6 Hz, H-6′b). ^13^C-NMR (150 MHz, D_2_O) δ: 64.59 (C-1), 105.35 (C-2), 84.72 (C-3), 80.60 (C-4), 86.38 (C-5), 64.04 (C-6), 65.41 (C-1′), 101.71 (C-2′), 79.78 (C-3′), 77.37 (C-4′), 84.11 (C-5′), 65.51 (C-6′). Comparison of the above data with those reported in the literature [14] showed they were in good agreement, confirming Compound **5** as *α*-D-Fructo*furanose*-1,2′:2,1′-*β*-D-Fructo*furanose* (DFA Ⅰ).

Compound **6**: White amorphous solid. HR-ESI-MS displayed a quasi-molecular ion peak at *m*/*z*: 341.1078 [M–H]^−^ (C_12_H_21_O_11_^−^, calculated 341.1073, error -1.665 ppm). ^1^H-NMR (600 MHz, D_2_O) *δ*: 5.42 (d, *J* = 4.2 Hz, H-1), 3.56 (dd, *J* = 4.2, 9.6 Hz, H-2), 3.76 (dd, *J* = 9.0, 9.6 Hz, H-3), 3.47 (dd, *J* = 9.0, 9.6 Hz, H-4), 3.85 (m, *J* = 3.0, 9.6 Hz, H-5), 3.82(dd, *J* = 3.0, 8.4 Hz, H-6a), 3.83 (dd, *J* = 3.6, 8.4 Hz, H-6b), 3.68 (d, *J* = 7.2 Hz, H-1′a, H-1′b), 4.22 (d, *J* = 8.4 Hz, H-3′), 4.06 (t, *J* = 8.4, 9.0 Hz, H-4′), 3.89 (m, *J* = 3.6, 6.0, 9.0 Hz, H-5′), 3.84 (d, *J* = 6.0 Hz, H-6′a, H-6′b). ^13^C-NMR (150 MHz, D_2_O) δ: 95.02 (C-1), 73.92 (C-2), 75.41 (C-3), 72.06 (C-4), 75.25 (C-5), 62.96 (C-6), 64.19 (C-1′), 106.53 (C-2′), 79.25 (C-3′), 76.83 (C-4′), 84.22 (C-5′), 65.21 (C-6′). Comparison of the above data with the NP-MRD database (https://np-mrd.org/) showed a high degree of consistency, confirming Compound **6** as sucrose.

**Figure 3:**
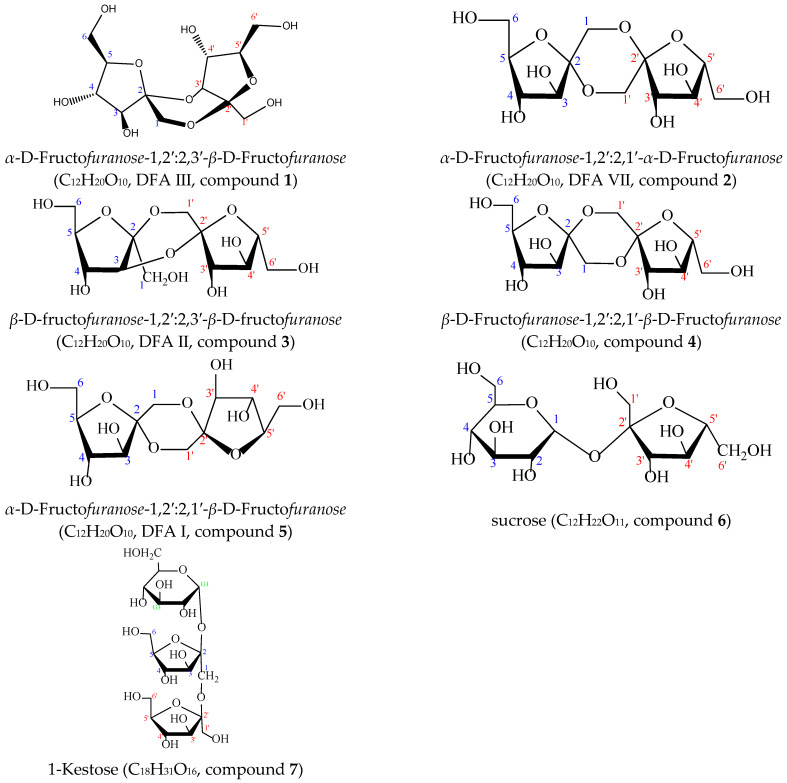
Structures and nomenclature of compounds **1**–**7**. The blue numbers indicate the carbon atom positions (C1–C6) on the fructose unit; the red numbers denote the carbon atom positions (C1′–C6′) on the fructose unit; the green numbers represent the carbon atom positions on the glucose unit.

Compound **7**: White amorphous solid. ESI-MS *m*/*z*: 503.1607 [M–H]^−^ (C_18_H_31_O_16_^−^, calculated 503.1613, error 1.309 ppm). ^1^H-NMR (600 MHz, D_2_O) *δ*: 5.44 (d, *J* = 4.2 Hz, G-H-1), 3.55 (dd, *J* = 4.2, 9.6 Hz, G-H-2), 3.75 (d, *J* = 9.6 Hz, G-H-3), 3.48 (t, *J* = 9.6 Hz, G-H-4), 3.82 (m, *J* = 3.0, 9.6 Hz, G-H-5), 3.80~3.83 (o, G-H-6a, G-H-6b), 3.72~3.75 (o, F-H-1a, F-H-1b), 4.20 (d, *J* = 9.0 Hz, F-H-3), 4.06 (dd, *J* = 5.4, 9.0 Hz, F-H-4), 3.88 (m, *J* = 5.4, 9.0 Hz, F-H-5), 3.75~3.87 (o, F-H-6a, F-H-6b), 3.69 (d, *J* = 12 Hz, F-H-1′a, F-H-1′b), 4.28 (d, *J* = 9.0 Hz, F-H-3′), 4.08 (dd, *J* = 8.4, 9.0 Hz, F-H-4′), 3.86 (m, *J* = 6.0, 9.0 Hz, F-H-5′), 3.75~3.87 (o, F-H-6′a, F-H-6′b). ^13^C-NMR (150 MHz, D_2_O) δ: 106.58 (G-C-1), 73.99 (G-C-2), 75.44 (G-C-3), 72.07 (G-C-4), 75.28 (G-C-5), 62.96 (G-C-6), 63.76 (F-C-1), 106.11 (F-C-2), 79.49 (F-C-3), 76.70 (F-C-4), 86.18 (F-C-5), 65.03 (F-C-6), 63.27 (F-C-1′), 106.58 (F-C-2′), 79.49 (F-C-3′), 77.32 (F-C-4′), 84.07 (F-C-5′), 65.18 (C-6′). Herein, G represents glucose, F represents fructose, and o denotes overlapping signals. The above data showed a high degree of consistency when compared with the NP-MRD database (https://np-mrd.org/) and the literature [16], confirming Compound **7** as 1-kestose. The structures of Compounds **1**–**7** are illustrated in Figure 3.

### 3.2. Quantitative Results of Difructose Anhydrides

**Figure 4:**
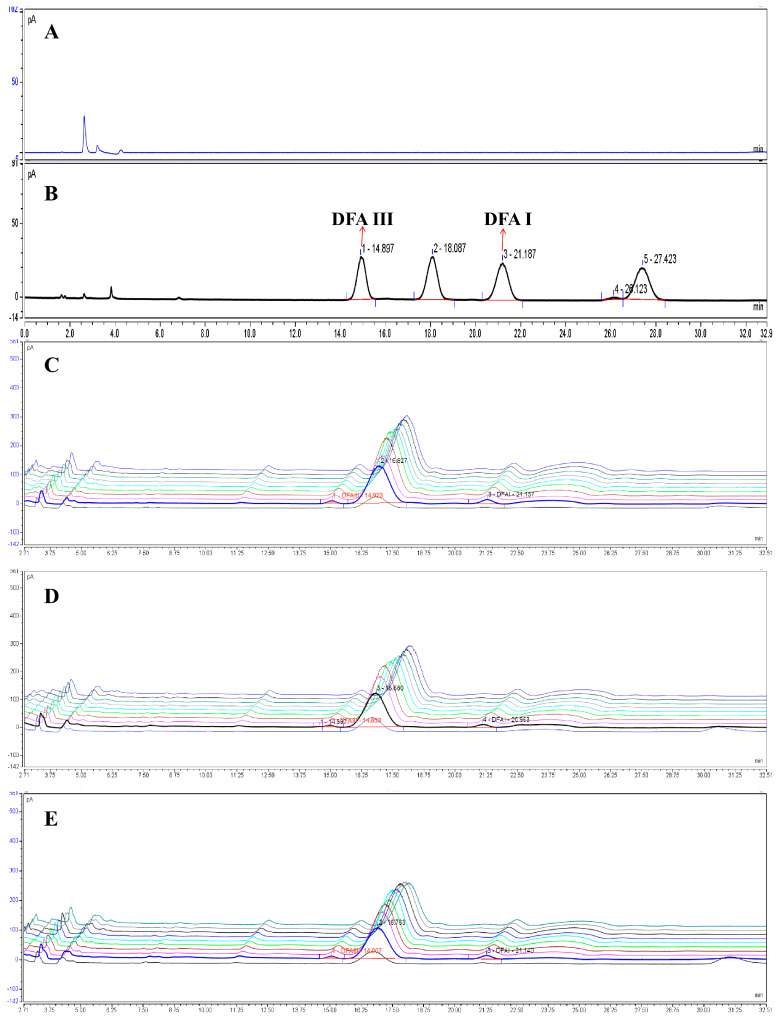
Column: XBridge BEH Amide Column (4.6 × 250 mm, 5 μm); Elution conditions: mobile phase A, acetonitrile; B, 0.1% ammonia solution; isocratic elution with 87% A–13% B; flow rate: 1.0 mL/min. (**A**) Chromatogram of the negative control. (**B**) Chromatogram of the mixed reference standards (compounds **1** and **5**). (**C**) LC chromatograms of samples K0–K9. (**D**) LC chromatograms of samples C0–C9. (**E**) LC chromatograms of samples S0–S9. Content results in sequence from front to back: raw Polygonati Rhizoma, once-steamed once-sun-dried, to nine-steamed nine-sun-dried samples.

Both the test solution and the reference solution were accurately pipetted at 10 μL each and injected into the liquid chromatography system for analysis. The chromatograms of the negative control, the mixed difructose anhydrides (DFAs) reference standards, and the PK, PC, and PS samples are shown in Figure 4.

#### 3.2.1. Method Validation for Difructose Anhydrides Content

(1) Linearity, Limits of Quantification (LOQs), and Limits of Detection (LODs): Aliquots of the reference standard solution prepared in Section 2.4.3 were accurately pipetted and gradually diluted with 80% ethanol to obtain a series of standard solutions at varying mass concentrations for Compound **1** (0.518, 0.259, 0.130, 0.065, 0.032, 0.016 mg/mL) and Compound **5** (0.523, 0.261, 0.131, 0.065, 0.033, 0.016 mg/mL). These solutions were then analyzed following the chromatographic conditions described in Section 2.4.1. The peak areas were recorded, and a linear regression was performed using the peak area (Y) as the ordinate and the mass concentration (X) as the abscissa. The resulting regression equations are presented in Table 2, with each standard curve demonstrating good linearity within its respective range. The reference standard solutions were further diluted stepwise for injection. The concentrations corresponding to signal-to-noise ratios (S/Ns) of 3:1 and 10:1 were defined as the LODs and LOQs, respectively. The LOD values for Compounds **1** and **5** are listed in Table 2.

(2) Precision test: Precisely 10 µL of the mixed reference solution from Section 2.4.3 was injected and analyzed under the conditions specified in Section 2.4.1. This was repeated for six consecutive injections. The RSD of the peak areas for compounds **1** and **5** from the six results were 0.60% and 0.86%, respectively, indicating good instrument precision (Table S2).

(3) Stability test: Precisely 10 µL of the K3 solution was injected at 0, 2, 4, 8, 16, and 24 h. The RSD of the peak areas from the six measurements was 1.11% for compound **1** and 0.76% for compound **5**, demonstrating good method stability (Table S2).

(4) Repeatability test: Six portions of K3 powder (0.5 g each) were taken. Test solutions were prepared according to Section 2.4.2 and analyzed under the conditions in Section 2.4.1. The peak areas were recorded. The average contents of compounds **1** and **5** were found to be 1.12% and 1.68%, with RSDs of 1.17% and 1.18%, respectively, indicating good method repeatability (Table S2).

(5) Recovery test: Six portions of K3 sample (0.25 g each) with known content were precisely weighed. Two reference solutions were precisely added to each portion. The mixtures were then prepared according to Section 2.4.2, injected, and the peak areas were recorded. The average recovery rates for compounds **1** and **5** were 98.86% and 98.14%, with RSDs of 1.29% and 1.14%, respectively (Table S2).

#### 3.2.2. Sample Analysis

**Figure 5:**
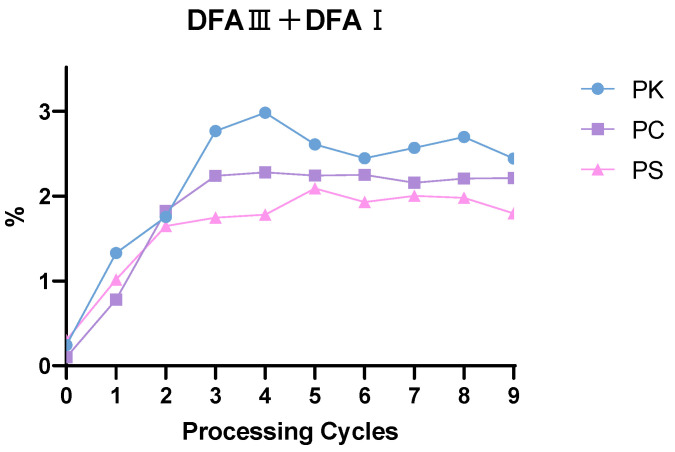
Changes in the content of DFA III and DFA I in Polygonati Rhizoma during the processing cycles.

Samples of raw and WPR were prepared according to the method described in Section 2.4.2. Their analysis was performed following the chromatographic conditions specified in Section 2.4.1 to determine the contents of two difructose anhydride components, DFA III and DFA I. The results are presented in Figure 5 and Table 3. The content determination results indicate a significant trend in the total content of DFA III and DFA I during the processing. After “three cycles of steaming and drying,” the total content of both compounds stabilized and reached their peak during the stages of “four cycles” to “five cycles of steaming and drying.” Specifically, starting from the third processing cycle, the total content of DFA III and DFA I ranged between 1.747% and 2.981%. In contrast, the total content in the raw material was less than 0.4%, demonstrating that the processing significantly promotes the accumulation of these two DFAs.

**Figure 6:**
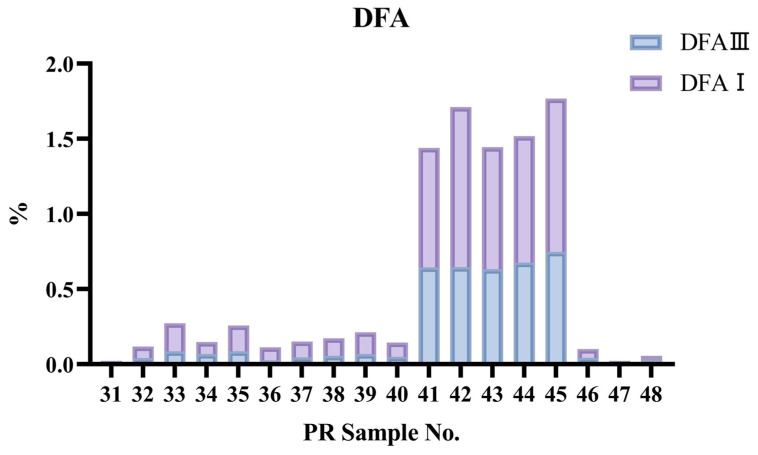
Results of DFAs content determination in market-sourced raw and wine-processed Polygonati Rhizoma samples.

The study also determined the contents of DFA III and DFA I in 18 batches of PR samples collected from the market. These samples included 13 batches of raw materials, comprising *Polygonatum kingianum*, *Polygonatum cyrtonema*, *Polygonatum sibiricum*, *Polygonatum hunanense*, *Polygonatum zanlanscianense* Pamp., *Polygonatum jinzhaiense*, and *Heteropolygonatum roseolum* M.N. Tamura et. Ogism, as well as 5 batches of WPR. The results, presented in Table 3 and Figure 6, show that the DFAs levels in the market samples are largely consistent with those in the laboratory-processed samples: the total content of DFA III and DFA I in all raw materials was below 0.3%, while in the processed products, the total content ranged from 1.44% to 1.77%, significantly higher than that in the raw materials. The combined content of DFA III and DFA I stabilizes after the 3rd to 4th round of steaming and sun-drying, and the pretreatment for their determination is straightforward.

### 3.3. Sensory Evaluation

A systematic sensory evaluation of raw and wine-processed PR samples at different processing stages was conducted by trained panelists. The evaluation focused on four dimensions: color, texture, odor, and taste, with detailed results presented in Table 4. The processing significantly influenced the sensory characteristics of WPR. In terms of color, the raw material exhibited a light brown hue. As the cycles of steaming and drying increased, the color progressively darkened, shifting from yellowish-brown to brown, then to dark brown, and finally turning black after the fourth cycle. The raw material was hard and difficult to break, with a faint odor. After processing, it initially became hard and brittle with a noticeable aroma, then transitioned to a soft and pliable texture. By the final stage, it displayed a soft yet tough consistency, accompanied by a slight burnt odor. The taste of the raw material was astringent and caused a pronounced numbing sensation on the tongue. During processing, this numbing sensation gradually diminished, while sweetness emerged and astringency faded. By the mid-to-late stages, the taste was predominantly sweet with a slight bitterness. At the final processing stage, a distinct sour and astringent taste appeared, along with a burnt flavor.

The sensory profile indicates a regular evolution in color, texture, odor, and taste of WPR throughout the “nine cycles of steaming and drying”. The overall characteristics shifted from “raw and astringent” to “cooked and mellow.” Notably, the appearance and taste after the fourth processing cycle aligned well with the traditional quality assessment criterion of “juice exhausted and color turning black, appearing glossy and pitch-black like lacquer, with a taste as sweet as maltose” described in the concept of “assessing quality by morphological characteristics.”

### 3.4. Determination of Chromaticity Values

**Figure 7:**
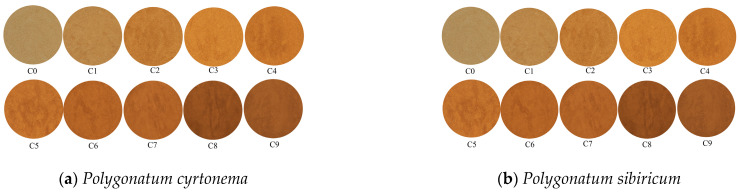
Color changes during processing.

The chromaticity values of WPR powder were quantified using a colorimeter. The appearance of the powdered samples is shown in Figure 7. The finely powdered raw and processed samples were evenly spread in a glass cuvette (2 × 4.5 × 1 cm) and measured in triplicate. The average values of L*, a*, b*, $E_{\mathrm{ab}}^{*}$, and ${\Delta E}_{\mathrm{ab}}^{*}$ were calculated. Here, L* represents lightness, ranging from 0 (black) to 100 (white); a* indicates the red–green component, with higher positive values denoting more red and higher negative values more green; b* indicates the yellow–blue component, with higher positive values denoting more yellow and higher negative values more blue. The total color difference of each sample was expressed as $E_{\mathrm{ab}}^{*}\;=\sqrt{L_{*}^{2}\;+\;a_{*}^{2}\;+\;b_{*}^{2}}$, and ΔE^*^_ab_ represents the degree of color difference between the sample and the raw material S0.

As shown in Table 5, the color of PR changed considerably from the raw state up to the fourth cycle of steaming and drying, and essentially stabilized after the sixth cycle. It is hypothesized that the chemical components related to color in PR also tended to stabilize by this stage.

The methodology for colorimetric measurement was validated. Fine powder of the PK sample K1 was evenly spread in a glass cuvette (2 × 4.5 × 1 cm) and measured six times consecutively. The recorded RSD values for L*, a*, and b* were 0.01%, 0.06%, and 0.05%, respectively, indicating good instrument precision. Six separate portions of the K1 fine powder were measured, and the RSD values for L*, a*, and b* were calculated to be 0.29%, 1.04%, and 0.64%, respectively, demonstrating good repeatability of the method. The fine powder of sample K1 was also measured at 0, 2, 4, 8, 16, and 24 h after being kept at room temperature. Each time point was measured six times, and the average values were used to calculate RSD of 0.38%, 1.69%, and 1.09% for L*, a*, and b*, respectively, confirming good sample stability within 24 h.

### 3.5. Reducing Sugar Content

Samples S0 to S9 of PR were prepared. Precisely 0.5 mL of each sample solution was drawn, and the standard curve preparation method described in Section 2.6.1 was followed starting from the step “adjusting to 4 mL with water”. The absorbance was measured at 540 nm, and the reducing sugar content in PR was calculated based on the standard curve. The results are shown in Table 6. With increasing processing time, the reducing sugar content increased from 4.71% to 5.71% in the raw materials to above 27.75% after being processed by “three cycles of steaming and drying”. This trend aligns closely with the scientific rationale behind the processing method. During the repeated cycles of steaming and drying, the macromolecular polysaccharides and glycosides in PR undergo hydrolysis, yielding a substantial amount of small-molecule reducing sugars such as glucose and fructose. Thus, the sharp increase in reducing sugar content serves as direct chemical evidence of the transformation in the material basis during the processing of PR, marking the progression of the procedure. It not only functions as a key quantitative indicator for evaluating the traditional “nine cycles of steaming and sun-drying” process but also provides a scientific explanation for the empirical observation that PR develops a sweeter taste after processing.

A methodological validation was performed for the determination of total reducing sugar content. The linear equation of the standard curve was Y = 17.538X − 0.1829. After blank subtraction, the absorbance values within the linear range were between 0.134 and 1.574, with an r value of 0.9989. The precision of the instrument was evaluated using K9 sample solution. The stability of the sample solution stored at room temperature was monitored at 0, 10, 30, 60, 90, and 150 min, yielding an RSD of 0.74% for the absorbance. The repeatability of the method was assessed using six parallelly prepared K9 sample solutions, and the RSD for the quantitative content was less than 2%. Furthermore, the accuracy was confirmed via a standard addition recovery experiment, yielding an average recovery rate for total reducing sugars of 97.85% with an RSD of 1.42% (n = 6). The detailed data mentioned above are provided in Table S3.

### 3.6. Total Polysaccharides and Total Oligosaccharides

Aliquots of 1 mL from the oligosaccharide test solution prepared in Section 2.7.3 were accurately transferred into separate 10 mL stoppered dry test tubes. Following the procedure described in Section 2.7.2 “Standard Curve Preparation,” starting from the step “add water to 2 mL”, the absorbance was measured according the anthrone–sulfuric acid method. Similarly, the polysaccharide test solution from Section 2.7.3 was accurately measured into a 10 mL stoppered dry test tube, and the absorbance was determined following the same procedure from “add water to 2 mL”. The results are presented in Table 6. The raw material exhibited the highest content of both total polysaccharides and total oligosaccharides. These contents decreased progressively during processing and stabilized after the “three cycles of steaming and drying” and “four cycles of steaming and drying”.

### 3.7. Determination of Free Sugar Content

#### 3.7.1. Methodological Validation

The methodological validation for the determination of free sugar content was conducted using HPLC. Following the method described in Section 2.8.3, “Preparation of Reference Standard Stock Solutions”, reference standards of fructose, glucose, sucrose, and 1-kestose were precisely weighed and serially diluted with 80% ethanol to prepare a series of standard solutions at different concentrations. Linear regression was performed by plotting the peak area (Y) against the mass concentration (X), yielding the following standard curve equations: Fructose: Y = 29.927X + 11.536, r = 0.9991; Glucose: Y = 28.749X + 2.3543, r = 0.9988; Sucrose: Y = 51.523X + 2.0467, r = 0.9981; 1-Kestose: Y = 34.783X + 1.7658, r = 0.9970. Each component exhibited a good linear relationship within its respective concentration range.

The LODs and the LOQs were determined based on S/Ns of 3:1 and 10:1, respectively, by injecting serially diluted standard solutions. The results were as follows: LODs for fructose, glucose, sucrose, and 1-kestose were 0.031, 0.06, 0.020, and 0.029 μg/mL, respectively; LOQs were 0.10, 0.20, 0.06, and 0.098 μg/mL, respectively.

The methodology validation, including assessments of precision, stability (at 0, 2, 4, 8, 16, and 24 h), repeatability, and spike recovery, showed that all results complied with the required standards. Detailed data are provided in Table S4.

#### 3.7.2. Content Determination Results

**Figure 8:**
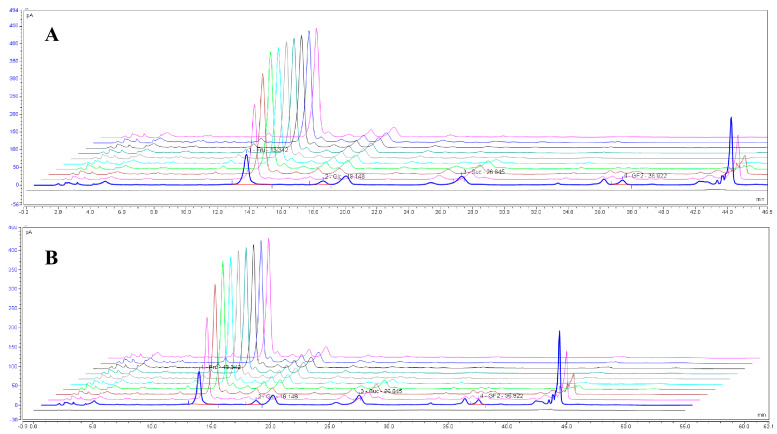
Chromatogram of wine-processed Polygonati Rhizoma (**A**) *Polygonatum cyrtonema*. (**B**) *Polygonatum sibiricum*. Column: Ecosil Grace Carbohydrate ES (5 μm, 250 × 4.6 mm); mobile phase A: acetonitrile, B: water; gradient: 0–18 min, 16–22% B; 18–22 min, 22% B; 28–30 min, 22–30% B; 30–35 min, 30% B; 35–40 min, 30–35% B; 41–47 min, 80% B; 48–55 min, 16% B. Flow rate: 1.0 mL/min; column temperature: 30 °C; injection volume: 10 μL. Content results in sequence from front to back: raw Polygonati Rhizoma, once-steamed once-sun-dried, to nine-steamed nine-sun-dried samples.

Samples of PR and WPR were prepared according to Section 2.8.2, and the contents of the four saccharide components were determined. The results are shown in Figure 8 and Table 6. During the processing, the contents of fructose and glucose increased, tending to stabilize after three cycles of steaming and drying and four cycles of steaming and drying, whereas the contents of sucrose and 1-kestose continuously decreased, becoming undetectable by the end of the processing.

### 3.8. Data Analysis

#### 3.8.1. Analysis of Chromaticity Data

**Figure 9:**
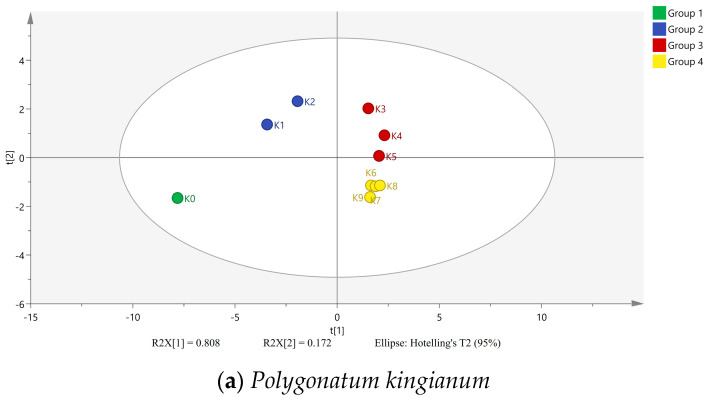

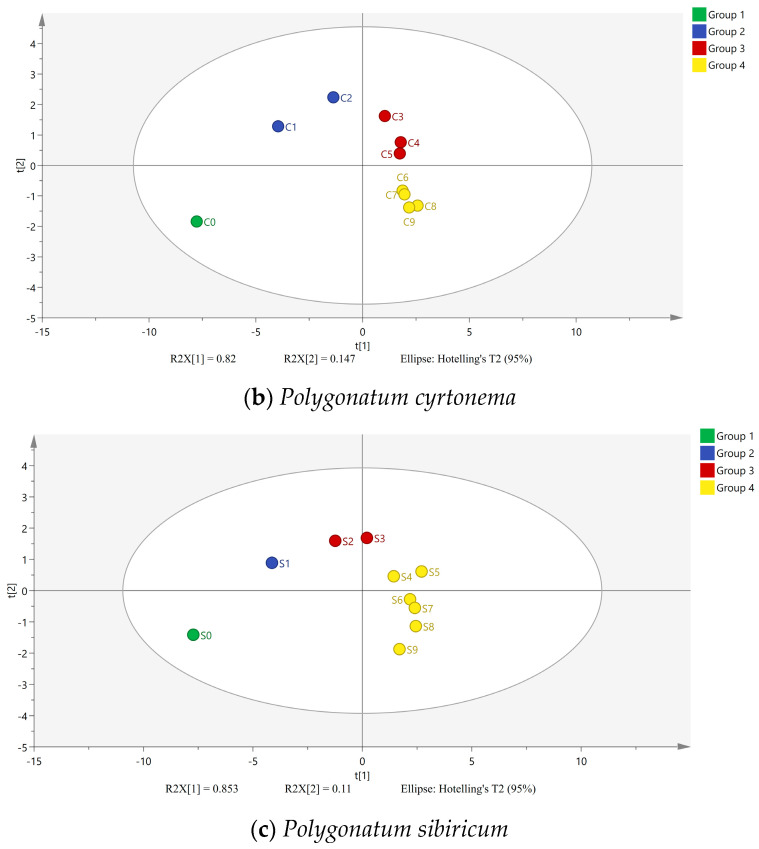
PCA plot of chromaticity and sugar content in raw and wine-processed Polygonati Rhizoma.

The chromaticity parameters (L*, a*, b*, and E^*^_ab_ values) along with the contents of DFAs, reducing sugars, polysaccharides, oligosaccharides, and free sugars for the raw and wine-processed PR samples were imported into the SIMCA 14.1 statistical software for unsupervised PCA. The results are presented in Figure 9. The PCA results indicate that the WPR samples from different steaming and drying cycles could be categorized into five groups. The raw material K0 formed a distinct group by itself. Samples K1 and K2 clustered together, located in the second quadrant. Samples K3 to K5 formed another cluster, distributed in the first quadrant. Samples K6 to K9 clustered together, located in the fourth quadrant. The data for PC and PS showed a similar pattern to that of PK.

#### 3.8.2. Correlation Analysis Between Color Changes and Sugar Content in PR

**Figure 10:**
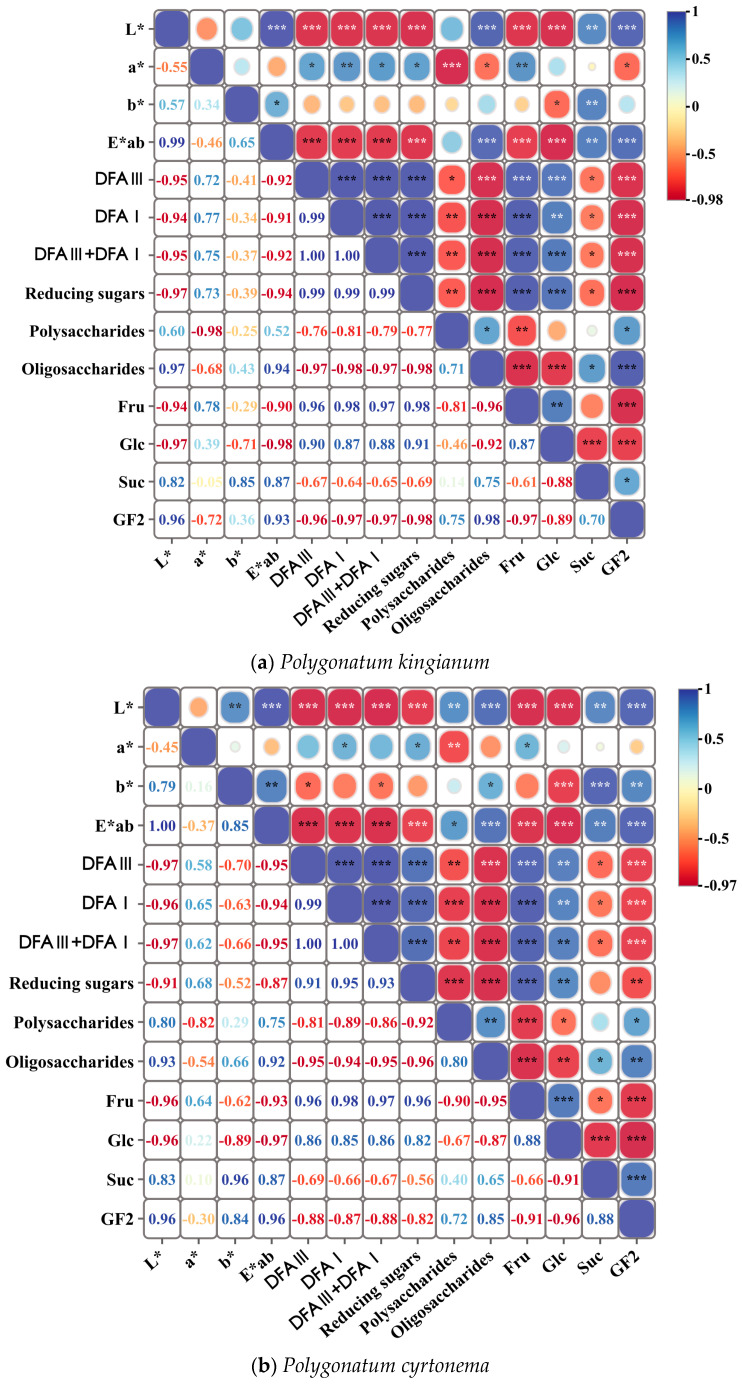

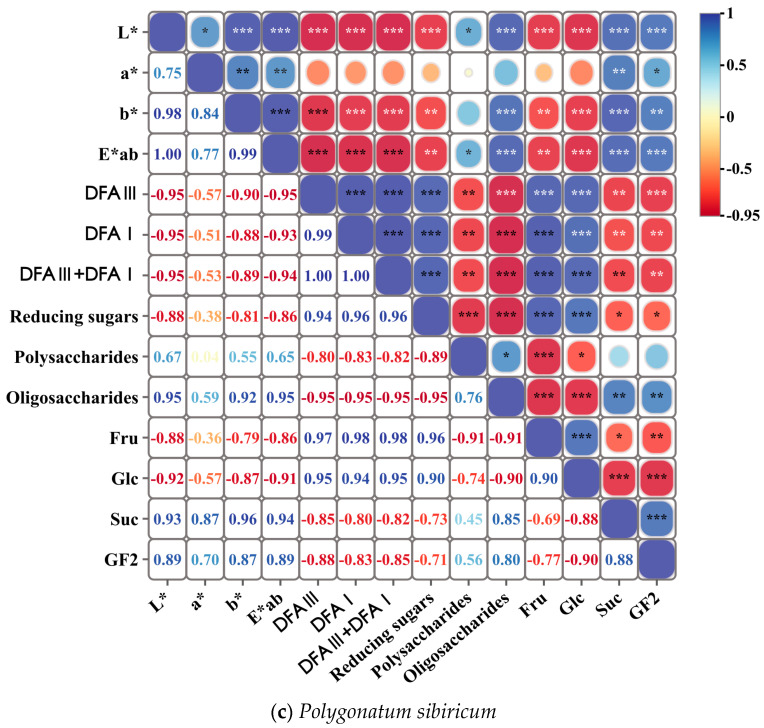
Heatmap of Pearson correlation analysis between chromaticity values and sugar content in raw and wine-steamed Polygonati Rhizoma. A value of −1 indicates a perfect negative correlation, while 1 indicates a perfect positive correlation. *** *p* < 0.001, ** *p* < 0.01, * *p* < 0.1.

To more intuitively analyze the correlation between the color and sugar content of WPR, a cluster heatmap analysis was performed using the powder chromaticity values (L*, a*, b*, E^*^_ab_) and the contents of various saccharide components in the raw and processed samples as indicators. As shown in Figure 10, the L* and E^*^_ab_ values showed a significant positive correlation (*p* < 0.01) with the contents of total oligosaccharides, sucrose, and 1-kestose, while exhibiting a significant negative correlation (*p* < 0.001) with the total content of DFA III and DFA I, total reducing sugars, fructose, and glucose. The a* value showed a significant positive correlation (*p* < 0.01) with the contents of fructose and DFA I, and a significant negative correlation (*p* < 0.001) with the total polysaccharide content. The b* value showed a significant positive correlation (*p* < 0.01) with the sucrose content. These results indicate that color changes during the processing of PR could potentially serve as an indicator for predicting changes in saccharide composition.

## 4. Discussion

This study systematically optimized the conditions for the content determination of DFA components, encompassing chromatographic conditions, detector parameters, and sample extraction methods.

To quantify DFAs, conditions were systematically optimized, covering chromatographic conditions, detector parameters, and sample extraction methods.

In the optimization of chromatographic conditions, the effects of column type, mobile phase composition, and column temperature on separation efficiency were investigated. Among the three columns tested—Ecosil Grace Carbohydrate ES, Shodex HILICpak VG-50 4E, and XBridge BEH Amide Column—the XBridge BEH Amide Column (4.6 × 250 mm, 5 μm) provided the best peak shapes for all analytes. To suppress mutarotation of sugars on the amide column, 0.1% ammonia solution was added to the aqueous phase, and the column temperature was appropriately increased. While higher temperature helps inhibit mutarotation [17], it compromised resolution; conversely, too low a temperature increased system pressure. Considering these factors, a column temperature of 30 °C was selected to balance resolution and system stability. For the optimization of CAD parameters, the effects of data acquisition rate (2, 5, 10, 20 Hz), filter constant (0.2, 0.5, 1, 2 s), and nebulizer temperature (30, 40, 50, 60 °C) on baseline noise and analyte response were evaluated. The final optimized parameters were a data acquisition rate of 5 Hz, a filter constant of 2.0 s, and a nebulizer temperature of 60 °C, which provided a higher response signal while effectively reducing baseline noise. Regarding sample extraction, considering the high polarity of saccharides, aqueous extraction is prone to microbial growth and co-extraction of macromolecular impurities like proteins and polysaccharides. This study systematically compared the effects of different ethanol concentrations (water, 40%, 80%, 95% ethanol), solvent volumes (20, 40, 60 mL), and ultrasonication times (15, 30, 45 min) on the extraction efficiency of the target components. The results (Figure S2) indicated that extraction with 80% ethanol under ultrasonication for 30 min provided sufficient extraction of the target DFAs and the highest efficiency for disaccharide components. Furthermore, while extraction yield increased with solvent volume, an excessively large volume could dilute target analytes to levels below the limit of quantitation. Therefore, 40 mL was ultimately selected as the optimal solvent volume.

In previous work, our research group established an analytical method for oligosaccharides in PR [6]. During the analysis of WPR, we observed an increased mass spectrometry response for a saccharide with a molecular weight of 324, which was speculated to be a DFA composed of two fructose units. As commercially available reference standards for DFAs are currently lacking, a series of isolation steps were performed on WPR to further elucidate its structure. This led to the isolation of seven soluble oligosaccharides. For the first time, a series of single-compound DFAs were isolated from WPR. The structures of five of these compounds were confirmed by high-resolution mass spectrometry and nuclear magnetic resonance spectroscopy. A method based on CAD was developed to determine the contents of two oligosaccharides, DFA III and DFA I, in WPR, with their contents found to exceed 1.4%. Although the contents of sucrose and total oligosaccharides change significantly during processing, their trends are consistently downward, making it difficult to directly reflect the transformation characteristics associated with “toxicity reduction and efficacy enhancement” in processing. Additionally, the determination of reducing sugars involves a relatively cumbersome procedure, which is not suitable for rapid detection. In contrast, the levels of DFA III and DFA I increase markedly after processing and show a significant correlation with changes in the color of the prepared slices. Therefore, they may be considered as potential quality markers for evaluating the degree of processing in WPR.

DFAs are a class of cyclic difructose derivatives, also regarded as potential prebiotics. Their structure is formed by the condensation of two fructose molecules through the removal of two water molecules and the formation of two interconnected glycosidic bonds. Due to variations in linkage positions, stereochemical configurations of fructose units, and other factors, DFAs exist in multiple isomeric forms [18]. It is widely reported in the literature that fructose and fructose-containing compounds can generate DFAs under non-enzymatic catalytic conditions [19] or via caramelization reactions during food processing. Furthermore, DFAs are also present in various higher plants [20]. This study found that raw PR contains trace amounts of DFAs as well. Based on their distribution and formation characteristics, DFAs have previously been proposed as quality markers for honey and coffee [21].

In terms of physiological functions, DFAs exhibit diverse biological activities. Among them, DFA III has been shown to promote the absorption of minerals such as calcium, iron, and zinc. Enhancing calcium absorption may help prevent osteoporosis, while improved iron absorption could be beneficial in preventing anemia. Additionally, DFA III can modulate bile acid metabolism by reducing the production of secondary bile acids, providing a basis for its potential role in preventing colon cancer [12]. On the other hand, DFA I promotes the production of short-chain fatty acids, particularly butyrate, and regulates gut microbiota by encouraging the growth of beneficial bacteria while inhibiting potential pathogens. These properties highlight its promising prospects as a functional food ingredient for promoting gastrointestinal health [22].

The significant increase in DFA III and DFA I after processing is consistent in direction with phenomena reported in some studies, where specific DFAs were generated in certain plant materials during heating or fermentation processes. However, this research was conducted primarily on a laboratory scale using a limited batch of PR raw material under relatively standardized conditions. While this approach clearly reveals the intrinsic patterns of chemical changes, it may not fully reflect all the variability introduced in industrial production due to factors such as different varieties, origins, harvest years of raw materials, and equipment differences. In subsequent work, the research team will further investigate the processing parameters, accumulate a sufficient quantity of DFA reference standards, and—based on traditional efficacy—conduct pharmacological activity studies and validation using model organisms.

## 5. Conclusions

Through systematic separation and identification of oligosaccharides in wine-steamed Polygonatum cyrtonema, five difructose anhydrides (DFAs) were obtained from this material for the first time. Analysis revealed that during processing, the contents of polysaccharides and oligosaccharides decreased, while reducing sugars such as fructose and glucose increased significantly. A strong correlation was observed between the colorimetric values of the processed slices and the changes in these carbohydrate components. These transformations may stem from the hydrolysis of polysaccharides and oligosaccharides, and the darkening of the slices is likely associated with the Maillard reaction between monosaccharides and amino acids. This provides a chemical perspective that aligns with the traditional processing description recorded in ancient texts: “the juice is depleted and the color turns black, appearing glossy like lacquer, with a taste as sweet as maltose.”

Quantitative analysis of the characteristic components DFA Ⅲ and DFA Ⅰ showed that their total content in raw materials was below 0.3%. Under controlled laboratory processing conditions, their content increased significantly and stabilized after the third to fourth rounds of steaming and drying, remaining consistently above 1.7% thereafter. Analysis of commercial samples further supported this trend: the total content of DFA Ⅲ and DFA Ⅰ in processed products (1.44–1.77%) was significantly higher than in all tested raw materials (<0.3%). These results indicate that the total content of DFA Ⅲ and DFA Ⅰ can serve as a chemical marker to distinguish between raw and processed Polygonatum cyrtonema and reflect the degree of processing.

However, the conclusions of this study have certain limitations. Firstly, the data were obtained on a laboratory scale using a limited number of batches under standardized conditions. While the threshold of “1.7%” is indicative within this experimental framework, its robustness against batch-to-batch variability in industrial production—caused by differences in raw materials, processing parameters, and environmental fluctuations—requires further statistical validation through larger-scale, multi-batch studies. Secondly, the CAD detection method established in this study, while superior to traditional methods in terms of sensitivity and pretreatment convenience, will undergo further external method validation and standardization research. This will assess its reproducibility across different laboratories, scalability, and suitability for rapid detection in actual production lines.

In summary, this study systematically elucidates the key transformation patterns of carbohydrate components during the processing of wine-steamed Polygonatum cyrtonema and preliminarily proposes the total content of DFA Ⅲ and DFA Ⅰ as a core potential indicator for evaluating the degree of processing. Future work should focus on the statistical validation of this indicator’s robustness, the standardization of the detection method, and research linking it to traditional pharmacological efficacy. This will advance its development into a scientific, reliable, and objective standard applicable for drug quality control and regulation.

## Figures and Tables

**Table 1:** The detailed information of Polygonati Rhizoma.

No.	Designation	Batch No.	Source
1	wine-processed Polygonati Rhizoma (J1)	TRT202301	Beijing Tongrentang Group
2	Raw *Polygonatum kingianum* (K0)	YNDH202401	Dehong, Yunnan
3	wine-processed *Polygonatum kingianum* (K1)	YNDH202402	Prepared in-house
4	wine-processed *Polygonatum kingianum* (K2)	YNDH202403	Prepared in-house
5	wine-processed *Polygonatum kingianum* (K3)	YNDH202404	Prepared in-house
6	wine-processed *Polygonatum kingianum* (K4)	YNDH202405	Prepared in-house
7	wine-processed *Polygonatum kingianum* (K5)	YNDH202406	Prepared in-house
8	wine-processed *Polygonatum kingianum* (K6)	YNDH202407	Prepared in-house
9	wine-processed *Polygonatum kingianum* (K7)	YNDH202408	Prepared in-house
10	wine-processed *Polygonatum kingianum* (K8)	YNDH202409	Prepared in-house
11	wine-processed *Polygonatum kingianum* (K9)	YNDH202410	Prepared in-house
12	Raw *Polygonatum cyrtonema* (C0)	AHBZ202401	Bozhou, Anhui
13	wine-processed *Polygonatum cyrtonema* (C1)	AHBZ202402	Prepared in-house
14	wine-processed *Polygonatum cyrtonema* (C2)	AHBZ202403	Prepared in-house
15	wine-processed *Polygonatum cyrtonema* (C3)	AHBZ202404	Prepared in-house
16	wine-processed *Polygonatum cyrtonema* (C4)	AHBZ202405	Prepared in-house
17	wine-processed *Polygonatum cyrtonema* (C5)	AHBZ202406	Prepared in-house
18	wine-processed *Polygonatum cyrtonema* (C6)	AHBZ202407	Prepared in-house
19	wine-processed *Polygonatum cyrtonema* (C7)	AHBZ202408	Prepared in-house
20	wine-processed *Polygonatum cyrtonema* (C8)	AHBZ202409	Prepared in-house
21	wine-processed *Polygonatum cyrtonema* (C9)	AHBZ202410	Prepared in-house
22	Raw *Polygonatum sibiricum* (S0)	HNLY202401	Luoyang, Henan
23	wine-processed *Polygonatum sibiricum* (S1)	HNLY202402	Prepared in-house
24	wine-processed *Polygonatum sibiricum* (S2)	HNLY202403	Prepared in-house
25	wine-processed *Polygonatum sibiricum* (S3)	HNLY202404	Prepared in-house
26	wine-processed *Polygonatum sibiricum* (S4)	HNLY202405	Prepared in-house
27	wine-processed *Polygonatum sibiricum* (S5)	HNLY202406	Prepared in-house
28	wine-processed *Polygonatum sibiricum* (S6)	HNLY202407	Prepared in-house
29	wine-processed *Polygonatum sibiricum* (S7)	HNLY202408	Prepared in-house
30	wine-processed *Polygonatum sibiricum* (S8)	HNLY202409	Prepared in-house
31	wine-processed *Polygonatum sibiricum* (S9)	HNLY202410	Prepared in-house
32	Raw *Polygonatum kingianum*	YNDL202501	Dali, Yunnan
33	Raw *Polygonatum cyrtonema*	AHJZ202401	Jinzhai, Anhui
34	Raw *Polygonatum zanlanscianense*	HBHA202401	Hongan, Hubei
35	Raw *Polygonatum jinzhaiense*	HBHA202402	Hongan, Hubei
36	Raw *Polygonatum hunanense*	HBES202401	Enshi, Hubei
37	Raw *Polygonatum sibiricum*	CQ200201	Chongqing
38	Raw *Polygonatum sibiricum*	HBA220901	Hubei
39	Raw Polygonati Rhizoma	HBA220501	Hubei
40	Raw Polygonati Rhizoma	HJ202401	Market-collected
41	Raw Polygonati Rhizoma	HN17021801	Hunan
42	wine-processed Polygonati Rhizoma	GZ190501	Guizhou
43	wine-processed Polygonati Rhizoma	GZ200201	Guizhou
44	wine-processed Polygonati Rhizoma	JX202401	Jiangxi
45	wine-processed Polygonati Rhizoma	HB191120	Hebei
46	wine-processed Polygonati Rhizoma	HNHY202401	Hengyang, Hunan
47	Raw *Heteropolygonatum roseolum*	SCLS202401	Leshan, Sichuan
48	Raw *Polygonatum kingianum*	YNDL202502	Dali, Yunnan
49	Raw *Polygonatum kingianum*	YNDL202503	Dali, Yunnan

**Table 2:** Linearity, LOQs, and LODs of the DFAs.

Compounds	Regression Equation	r	Linear Range (μg/mL)	LOQ (μg/mL)	LOD (μg/mL)
DFA Ⅲ	Y = 53.697X + 2.1644	0.9986	4.8~1036.0	4.80	1.16
DFA Ⅰ	Y = 60.473X + 2.2758	0.9990	7.63~1045.0	7.63	2.05

**Table 3:** Determination results of DFAⅢ and DFAⅠ content.

No.	Designation	DFA Ⅲ (%)	DFA Ⅰ (%)	DFA Ⅲ + DFA Ⅰ (%)
1	Raw PR	0.08	0.17	0.24
2	wine-processed PR	0.44	0.89	1.33
3	wine-processed PR	0.62	1.13	1.75
4	wine-processed PR	1.07	1.70	2.77
5	wine-processed PR	1.16	1.82	2.98
6	wine-processed PR	1.01	1.60	2.61
7	wine-processed PR	0.93	1.51	2.45
8	wine-processed PR	0.99	1.58	2.57
9	wine-processed PR	1.06	1.64	2.70
10	wine-processed PR	0.93	1.51	2.44
11	Raw PR	0.01	0.09	0.10
12	wine-processed PR	0.21	0.57	0.78
13	wine-processed PR	0.70	1.12	1.83
14	wine-processed PR	0.93	1.31	2.24
15	wine-processed PR	0.95	1.33	2.28
16	wine-processed PR	0.94	1.31	2.24
17	wine-processed PR	0.93	1.32	2.25
18	wine-processed PR	0.88	1.28	2.16
19	wine-processed PR	0.90	1.30	2.21
20	wine-processed PR	0.91	1.30	2.21
21	Raw PR	0.11	0.20	0.30
22	wine-processed PR	0.33	0.69	1.02
23	wine-processed PR	0.58	1.06	1.65
24	wine-processed PR	0.66	1.09	1.75
25	wine-processed PR	0.64	1.14	1.78
26	wine-processed PR	0.80	1.29	2.09
27	wine-processed PR	0.74	1.19	1.93
28	wine-processed PR	0.76	1.24	2.00
29	wine-processed PR	0.74	1.24	1.98
30	wine-processed PR	0.67	1.13	1.80
31	Raw PR	0.00	0.02	0.02
32	Raw PR	0.04	0.08	0.12
33	Raw PR	0.09	0.19	0.27
34	Raw PR	0.07	0.08	0.15
35	Raw PR	0.09	0.17	0.26
36	Raw PR	0.03	0.09	0.11
37	Raw PR	0.05	0.11	0.15
38	Raw PR	0.06	0.12	0.17
39	Raw PR	0.07	0.15	0.21
40	Raw PR	0.05	0.10	0.15
41	wine-processed PR	0.64	0.80	1.44
42	wine-processed PR	0.65	1.07	1.71
43	wine-processed PR	0.63	0.81	1.45
44	wine-processed PR	0.68	0.84	1.52
45	wine-processed PR	0.75	1.02	1.77
46	Raw PR	0.04	0.06	0.10
47	Raw PR	0.00	0.02	0.02
48	Raw PR	0.03	0.03	0.06

**Table 4:** Evaluation of Appearance Characteristics of Raw and Wine-processed wine-processed Polygonati Rhizoma at different processing stages (0–9).

No.	Color	Texture	Odor	Taste
0	light brown	hard and difficult to break	faint scent	astringent with strong numbing
1	yellowish-brown	hard and brittle	slightly aromatic	slight numbing
2	brown	moderately hard	slightly aromatic	slightly sweet, somewhat astringent
3	dark brown	soft	aromatic	sweet, slightly astringent
4	black	soft yet resistant to breaking	aromatic	sweet, no astringency
5	black	soft and sticky	fragrant	sweet, slightly bitter
6	jet-black	soft and pliable	strong scorched aroma	sweet, slightly bitter
7	jet-black	soft and pliable	scorched aroma	mildly sweet, somewhat bitter
8	jet-black	soft and pliable	slightly burnt odor	sour and bitter
9	jet-black	soft and pliable	slightly burnt odor	heavily sour-astringent with charred aftertaste

**Table 5:** Determination results of color parameters for powdered PC, PS, and their processed products (n = 3).

Sample	L*	a*	b*	$E_{\mathrm{ab}}^{*}$	${\Delta E}_{\mathrm{ab}}^{*}$ (vs. Raw Material)
C0	80.11 ± 0.00	5.36 ± 0.01	21.35 ± 0.01	83.080 ± 0.00	0
C1	70.18 ± 0.01	9.07 ± 0.01	25.70 ± 0.01	75.28 ± 0.00	17.64 ± 0.01
C2	61.99 ± 0.01	10.72 ± 0.01	23.69 ± 0.01	67.23 ± 0.01	24.31 ± 0.00
C3	55.45 ± 0.01	10.67 ± 0.01	19.97 ± 0.01	59.89 ± 0.01	29.64 ± 0.01
C4	52.92 ± 0.01	10.26 ± 0.01	17.51 ± 0.03	56.68 ± 0.02	31.79 ± 0.01
C5	51.94 ± 0.00	9.85 ± 0.02	16.41 ± 0.01	55.35 ± 0.00	32.64 ± 0.00
C6	49.94 ± 0.02	9.110 ± 0.01	14.28 ± 0.01	52.74 ± 0.02	34.53 ± 0.02
C7	50.64 ± 0.01	8.66 ± 0.01	14.47 ± 0.01	53.37 ± 0.01	33.76 ± 0.01
C8	49.25 ± 0.01	7.93 ± 0.01	13.03 ± 0.02	51.56 ± 0.01	35.13 ± 0.01
C9	49.42 ± 0.01	7.92 ± 0.01	13.22 ± 0.01	51.76 ± 0.01	34.94 ± 0.01
S0	68.57 ± 0.01	9.23 ± 0.01	24.66 ± 0.01	73.45 ± 0.01	0
S1	61.57 ± 0.00	9.83 ± 0.01	20.48 ± 0.01	65.63 ± 0.00	23.66 ± 0.00
S2	56.84 ± 0.01	10.08 ± 0.02	19.20 ± 0.01	60.83 ± 0.01	28.07 ± 0.01
S3	54.20 ± 0.01	9.31 ± 0.01	16.58 ± 0.01	57.44 ± 0.01	30.32 ± 0.01
S4	50.627 ± 0.01	8.48 ± 0.01	13.79 ± 0.02	53.15 ± 0.02	33.78 ± 0.01
S5	49.77 ± 0.02	8.14 ± 0.01	12.93 ± 0.02	52.07 ± 0.02	34.65 ± 0.02
S6	49.30 ± 0.01	7.65 ± 0.01	12.47 ± 0.01	51.42 ± 0.01	35.10 ± 0.01
S7	48.82 ± 0.01	7.23 ± 0.01	11.77 ± 0.02	50.74 ± 0.00	35.60 ± 0.00
S8	48.04 ± 0.02	6.56 ± 0.02	10.75 ± 0.02	49.66 ± 0.02	36.45 ± 0.01
S9	47.47 ± 0.01	6.12 ± 0.01	10.16 ± 0.01	48.93 ± 0.01	37.07 ± 0.01

**Table 6:** Sugar content of raw and wine-processed Polygonati Rhizoma (n = 2). — indicates below the limit of detection.

Sample	Content (%)						
	Total Reducing Sugar	Total Polysaccharide	Total Oligosaccharide	Fructose	Glucose	Sucrose	1-Kestose
C0	5.71	10.18	54.99	4.49	0.42	4.41	1.46
C1	25.01	4.70	44.55	17.36	2.08	4.67	0.93
C2	32.18	3.92	33.21	20.71	2.02	4.32	1.00
C3	37.71	3.68	23.18	28.00	3.56	3.57	0.30
C4	36.58	3.64	21.24	29.81	4.71	2.12	0.19
C5	38.86	4.07	21.57	28.40	5.13	1.96	0.40
C6	30.87	4.01	31.03	27.58	5.29	0.13	—
C7	37.12	4.30	26.04	27.41	5.26	—	—
C8	40.40	4.17	17.15	28.34	6.06	—	—
C9	35.69	4.08	24.50	27.11	5.71	—	—
S0	4.71	8.52	71.01	4.80	0.21	2.56	1.48
S1	21.00	5.79	51.86	14.66	0.85	2.43	2.10
S2	23.62	4.73	48.59	22.04	2.14	2.17	0.59
S3	27.75	3.15	43.19	23.74	2.48	1.57	0.57
S4	28.53	3.85	37.27	22.36	3.36	0.59	—
S5	33.46	3.59	37.63	24.94	4.01	—	—
S6	30.29	4.63	31.15	23.14	3.05	—	—
S7	28.56	4.86	37.26	24.20	3.76	—	—
S8	29.13	5.33	34.88	22.72	3.35	—	—
S9	24.00	5.53	40.16	20.27	2.57	—	—

## Data Availability

The datasets used and analyzed during the current study are available from the corresponding authors on reasonable request.

## Supplementary Materials

The following supporting information can be downloaded at: https://www.mdpi.com/article/10.3390/foods15030584/s1, Table S1: 1H and 13C NMR spectral data of compound (600/150 MHz, D2O, ppm); Table S2: Stability, repeatability, precision and recovery of 2 DFAs; Table S3: Stability, repeatability, precision and recovery of reducing sugar; Table S4: Stability, repeatability, precision and recovery of free sugars; Figure S1: The 1H and 13C NMR spectra of compounds **1**–**7**; Figure S2: Extraction efficiency of two DFAs under different conditions; (A) Sonication time; (B) Extraction solvent. (C) Solvent volume.

## References

[1] Pharmacopoeia Commission Executive Committee (2025). Pharmacopoeia of the People’s Republic of China.

[2] Wang S.Y., Zhou N., Shi N.X., Zhang G.F., Liu H.Y., Guo X.R., Ji Y.H. (2023). Testing and using complete plastomes for authentication of medicinal *Polygonatum* species (Asparagaceae). Ind. Crops Prod..

[3] Jiang T. (2023). Study on the Processing Chemistry in Polygonatum cyrtonema Hua and the Enhancement Mechanism of Processing.

[4] Zhao P., Zhao C.C., Li X., Gao Q.Z., Huang L.Q., Xiao P.G., Gao W.Y. (2018). The genus *Polygonatum*: A review of ethnopharmacology, phytochemistry and pharmacology. J. Ethnopharmacol..

[5] Zheng X.Q., Jin C.S., Zhang Y.Z., Liu J.L., Li L.X., Feng Q. (2020). Dynamic changes of saccharides from *Polygonatum cyrtonema* during Nine-Steam-Nine-Bask processing. Chin. Tradit. Pat. Med..

[6] Guo H., Yao R., Fan J., Wang Y., Zhang L., Sun H., Guo X., Yang J., Pu J., Zhang Y. (2025). Profiling oligosaccharide components in *Polygonatum kingianum* with potential anti-NAFLD activity using UPLC-Orbitrap-MS/MS technology. Food Hydrocoll. Health.

[7] He L.L., Yan B.X., Yao C.Y., Chen X.Y., Li L.W., Wu Y.J., Song Z.J., Song S.S., Zhang Z.F., Luo P. (2021). Oligosaccharides from *Polygonatum Cyrtonema* Hua: Structural characterization and treatment of LPS-induced peritonitis in mice. Carbohydr. Polym..

[8] Guan Y., Li R., Lv Z., Bo N., Wang T., Guo Y., Liang Z., Zhang J., Fan Q., Dang L. (2026). The change in structure and improvement of anti-aging effects of polysaccharides in Polygonati Rhizoma after the traditional “Nine Steaming-Nine Sun-Drying”. Carbohydr. Polym..

[9] Du L., Nong M.N., Zhao J.M., Peng X.M., Zong S.H., Zeng G.F. (2016). *Polygonatum sibiricum* polysaccharide inhibits osteoporosis by promoting osteoblast formation and blocking osteoclastogenesis through Wnt/β-catenin signalling pathway. Sci. Rep..

[10] Cai J.L., Zhu Y.L., Zuo Y.J., Tong Q.Z., Zhang Z.G., Yang L., Li X.P., Yi G.Q. (2019). *Polygonatum sibiricum* polysaccharide alleviates inflammatory cytokines and promotes glucose uptake in high-glucose- and high-insulin-induced 3T3-L1 adipocytes by promoting Nrf2 expression. Mol. Med. Rep..

[11] Mellet C.O., García Fernández J.M. (2010). Difructose dianhydrides (DFAs) and DFA-enriched products as functional foods. Top. Curr. Chem..

[12] Cheng M., Wu H., Zhang W., Mu W. (2022). Difructose anhydride III: A 50-year perspective on its production and physiological functions. Crit. Rev. Food Sci. Nutr..

[13] (2015). People’s Republic of China Agricultural Professional Standard. Determination of Soluble Sugar in Fruits and Derived Products 3,5-Dinitrosalicylic Acid Colorimetry.

[14] Trabs K., Kasprick N., Henle T. (2011). Isolation and identification of Di-D-fructose dianhydrides resulting from heat-induced degradation of inulin. Eur. Food Res. Technol..

[15] Blize A.E., Manleyharris M., Richards G.N. (1994). Di-d-fructose dianhydrides from the pyrolysis of inulin. Carbohydr. Res..

[16] Duan W., Wei Y., Chen Z., Wang D., Wang X., Geng Y. (2017). Preparation of kestose certified reference materials. Food Res. Dev..

[17] Liu J.Y., Li J.W., Wang C. (2017). Determination of monosaccharide composition in cellulose hydrolysate during the production of levulinic acid from biomass by high performance liquid chromatography with charged aerosol detection. Chem. Bioeng..

[18] Wang X., Yu S.H., Zhang T., Jiang B., Mu W.M. (2015). From fructans to difructose dianhydrides. Appl. Microbiol. Biotechnol..

[19] Christian T.J., Manley-Harris M., Field R.J., Parker B.A. (2000). Kinetics of formation of di-D-fructose dianhydrides during thermal treatment of inulin. J. Agric. Food Chem..

[20] Li H.Y., Hagiwara H., Zhu W.R., Yokoyama C., Harada N. (1997). Isolation and NMR studies of di-D-fructose anhydride III from Lycoris radiata Herbert by supercritical extraction with carbon dioxide. Carbohydr. Res..

[21] Montilla A., Ruiz-Matute A.I., Sanz M.L., Martínez-Castro I., del Castillo M.D. (2006). Difructose anhydrides as quality markers of honey and coffee. Food Res. Int..

[22] Yu S.H., Li Q.T., Wang Z.L., Zhu M.Y. (2025). In Vitro Physiological Properties of Difructose Anhydride I Prepared from Inulin by Inulin Fructotransferase. J. Agric. Food Chem..

