# Supplementary material for: Study on the Separation, Identification, and Quality Control Methods of Oligosaccharide Components in Wine-Processed Polygonati Rhizoma
Source: Foods. 2026 Feb 5;15(3):584. doi: 10.3390/foods15030584 (PMC12896846; doi:10.3390/foods15030584)
Supplement: Supplementary file 1 [file foods-4071138-supplementary.pdf › foods-4071138-supplementary.pdf]

Supplementary materials

Study on the Separation, Identification, and Quality Control Methods of Oligosaccharide Components in Wine-processed Polygonati Rhizoma

Content

Table S1 1H and 13C NMR spectral data of compound (600/150 MHz, D_2O, ppm) 2

Table S2. Stability, repeatability, precision and recovery of 2 DFAs. 3

Table S3. Stability, repeatability, precision and recovery of reducing sugar. 4

Table S4. Stability, repeatability, precision and recovery of free sugars. 4

Figure S1 The 1H and 13C NMR spectra of compounds **1-7** 12

Figure S2 Extraction efficiency of two DFAs under different conditions (A) Sonication time (B) Extraction solvent (C) Solvent volume 13

Table S1 1H and 13C NMR spectral data of compound **1-7**(600/150 MHz, D_2O, ppm)

Position	Compound **1**		Compound **2**		Compound **3**		Compound **4**		Compound **5**		Compound **6**		Compound **7**	
	^{1}H (*J* in Hz)	δ_C	^{1}H (*J* in Hz)	δ_C	^{1}H (*J* in Hz)	δ_C	^{1}H (*J* in Hz)	δ_C	^{1}H (*J* in Hz)	δ_C	^{1}H (*J* in Hz)	δ_C	^{1}H (*J* in Hz)	δ_C
1	4.06,d(13.2); 3.77,d(13.2)	61.81	4.02,d(12.6); 3.84,d(12.6)	63.94	4.04,d(12.0); 3.76,d(12.0)	66.23	4.03,d(13.2); 3.96,d(12.6)	66.40	4.16,d(12.0); 3.70,d(12.0)	64.59	5.42,d(4.2)	95.02	3.72~3.75(o)	63.76
2		106.43		106.65		106.53		103.49		105.35	3.56,dd(4.2, 9.6)	73.92		106.11
3	4.16,d(4.2)	84.01	4.06,d(3.6)	82.81	4.20,d(1.2)	76.16	4.02, d(7.8)	81.96	4.05,d(2.4)	84.72	3.76,dd(9.0, 9.6)	75.41	4.20, d (9.0)	79.49
4	3.89,dd(1.2, 4.2)	75.30	3.97,dd(3.6, 6.0)	79.63	4.10,dd(1.2, 2.4)	78.78	4.10, dd(1.2, 7.8)	77.42	3.96,dd(2.4, 5.4)	80.60	3.47,dd(9.0, 9.6)	72.06	4.06, t(5.4, 9.0)	76.70
5	4.11,m(1.2, 3.0,3.6)	84.71	4.02,m(3.0, 6.0)	85.80	3.98,m(2.4, 5.4, 6.0)	87.51	3.89, m(1.2, 3.6, 6.6)	84.25	4.03,m(3.0, 5.4)	86.38	3.85,m(3.0, 9.6)	75.25	3.88,m(5.4,9.0)	86.18
6	3.90,dd(3.6, 12.6); 3.82,dd(3.0, 12.6)	63.92	3.82,dd(3.0, 12.6); 3.70,dd(6.0, 12.6)	63.84	3.76,dd(6.0, 12.6); 3.75,dd(5.4, 12.6)	65.02	3.78,dd(3.6, 12.6); 3.66,dd(6.6, 12.6)	65.20	3.87,dd(3.0, 12.6); 3.75,dd(5.4, 12.6)	64.04	3.82,dd(3.0,8.4); 3.83,dd(3.6,8.4)	62.96	3.75~3.87(o)	65.03
1′	3.71,d(12.0); 3.65,d(12.0)	66.27	4.02,d(12.6); 3.84,d(12.6)	63.94	3.71,d(12.6); 3.67,d(12.6)	65.07	4.03,d(13.2); 3.96,d(12.6)	66.40	4.23,d(12.0); 3.62,d(12.0)	65.41	3.68, d (7.2)	64.19	3.69, d (12)	63.27
2′		104.19		106.65		100.76		103.49		101.71		106.53		106.58
3′	4.34,d(7.0)	81.86	4.06,d(3.6)	82.81	3.88,d(7.8)	79.90	4.02,d(7.8)	81.96	3.88,d(8.4)	79.78	4.22, d (8.4)	79.25	4.28, d (9.0)	79.49
4′	4.67,dd(1.2, 7.0)	72.19	3.97,dd(3.6, 6.0)	79.63	4.14,dd(7.2, 7.8)	77.03	4.10,dd(1.2, 7.8)	77.42	4.13,dd(7.2, 8.4)	77.37	4.06, t (8.4, 9.0)	76.83	4.08, t (8.4, 9.0)	77.32
5′	3.71,m(1.2, 4.8,5.4)	82.97	4.02,m(3.0, 6.0)	85.80	3.91,m(3.6, 6.6, 7.2)	83.99	3.89,m(1.2, 3.6, 6.6)	84.25	3.94,m(3.0, 6.6, 7.2)	84.11	3.89,m(3.6, 6.0, 9.0)	84.22	3.86, m(6.0, 9.0)	84.07

6′	3.76,dd(4.8, 12.6); 3.65,dd(5.4, 12.6)	63.43	3.82,dd(3.0, 12.6); 3.70,dd(6.0, 12.6)	63.84	3.80,dd(3.6, 12.6); 3.68,dd(6.6, 12.6)	65.43	3.78,dd(3.6, 12.6); 3.66,dd(6.6, 12.6)	65.20	3.80,dd(3.0, 12.6); 3.62,dd(6.6, 12.6)	65.51	3.84, d (6.0)	65.21	3.75~3.87(o)	65.18
G1													5.44, d (4.2)	106.58
2													3.55, dd (4.2, 9.6)	73.99
G3													3.75, d (9.6)	75.44
4													3.48, t (9.6)	72.07
5													3.82, m (3.0, 9.6)	75.28
6													3.80~3.83(o)	62.96

Table S2. Stability, repeatability, precision and recovery of DFA III and DFA I.

	Precision		Stability		Repeatability		Recovery					
Name	Peak area	RSD/% (n=6)	Peak area	RSD/% (n=6)	Content/%	RSD/% (n=6)	Original/mg	Added/mg	Found/mg	Recovery/%	Average value/%	RSD/% (n=6)
DFA III	13.243	0.60	10.147	1.11	1.139	1.17	2.810	2.037	4.826	98.94	98.86	1.29
	13.203		10.348		1.134		2.800	2.037	4.845	100.39		
	13.239		10.343		1.133		2.810	2.037	4.823	98.81		
	13.298		10.184		1.108		2.800	2.037	4.832	99.72		
	13.377		10.338		1.110		2.815	2.037	4.825	98.67		
	13.397		10.096		1.125		2.819	2.037	4.788	96.63		
DFA I	15.627	0.86	15.492	0.76	1.647	1.18	4.215	3.580	7.765	99.17	98.14	1.14
	15.648		15.714		1.699		4.200	3.580	7.714	98.17		

	15.408		15.496		1.668		4.215	3.580	7.673	96.59		
	15.661		15.505		1.682		4.200	3.580	7.679	97.19		
	15.791		15.453		1.664		4.222	3.580	7.738	98.21		
	15.754		15.356		1.695		4.229	3.580	7.791	99.50		

Table S3. Stability, repeatability, precision and recovery of reducing sugar.

	Stability			Repeatability		Precision		Recovery					
Name	Time/min	Absorbance	RSD/% (n=6)	Content/%	RSD/% (n=6)	Absorbance	RSD/% (n=6)	Original/mg	Added/mg	Found/mg	Recovery/%	Average value/%	RSD/% (n=6)
reducing sugar	0	0.507	0.74	41.787	1.11	0.474	0.09	0.221	0.252	0.462	95.70	97.85	1.42
	10	0.512		42.772		0.474		0.224	0.252	0.475	99.66		
	30	0.518		42.181		0.474		0.224	0.252	0.471	98.08		
	60	0.513		42.139		0.474		0.223	0.252	0.472	98.87		
	90	0.514		42.280		0.474		0.220	0.252	0.466	97.74		
	150	0.516		43.076		0.473		0.223	0.252	0.467	97.06		

Table S4. Stability, repeatability, precision and recovery of free sugars.

	Stability		Repeatability		Precision		Recovery					
Name	Peak area	RSD/% (n=6)	Content/%	RSD/% (n=6)	Peak area	RSD/% (n=6)	Original/mg	Added/mg	Found/mg	Recovery/%	Average value/%	RSD/% (n=6)
Fructose	27.173	2.26	5.465	1.79	99.816	1.01	2.740	2.769	5.727	96.19	98.87	3.04
	26.796		5.603		98.619		2.814	2.769	5.836	95.66		
	27.597		5.623		99.179		2.820	2.769	5.758	97.07		
	28.464		5.561		99.444		2.797	2.769	5.557	100.15		
	26.825		5.708		100.505		2.856	2.769	5.570	100.99		

	27.324		5.743		101.573		2.881	2.769	5.476	103.16		
	10.670		2.711		19.670		1.324	1.391	2.805	96.77		
	10.444		2.815		19.505		1.359	1.391	2.802	98.14		
Glucose	10.569	2.18	2.812	2.54	19.600	0.66	1.414	1.391	2.723	102.96	101.47	3.18
	10.883		2.763		19.578		1.410	1.391	2.732	102.52		
	10.196		2.889		19.647		1.390	1.391	2.679	103.78		
	10.620		2.896		19.910		1.446	1.391	2.709	104.67		
	27.168		3.916		33.342		2.113	2.545	4.845	96.143		
	28.679		4.041		33.680		2.029	2.545	4.669	97.979		
Sucrose	27.764	2.46	4.083	2.41	33.734	0.92	2.048	2.545	4.588	100.104	101.26	3.72
	28.109		4.045		34.013		2.035	2.545	4.423	103.541		
	27.156		4.188		34.120		2.096	2.545	4.451	104.267		
	28.679		4.165		34.249		2.089	2.545	4.390	105.555		
	5.168		1.048		12.541		0.525	0.527	1.093	96.20		
	5.298		1.076		12.567		0.540	0.527	1.047	101.89		
Kestose	5.430	2.50	1.067	1.49	12.700	1.45	0.535	0.527	1.040	102.06	100.88	3.92
	5.430		1.061		12.726		0.534	0.527	1.013	104.68		
	5.165		1.093		12.845		0.547	0.527	1.026	104.61		
	5.449		1.082		12.965		0.543	0.527	1.116	95.84		

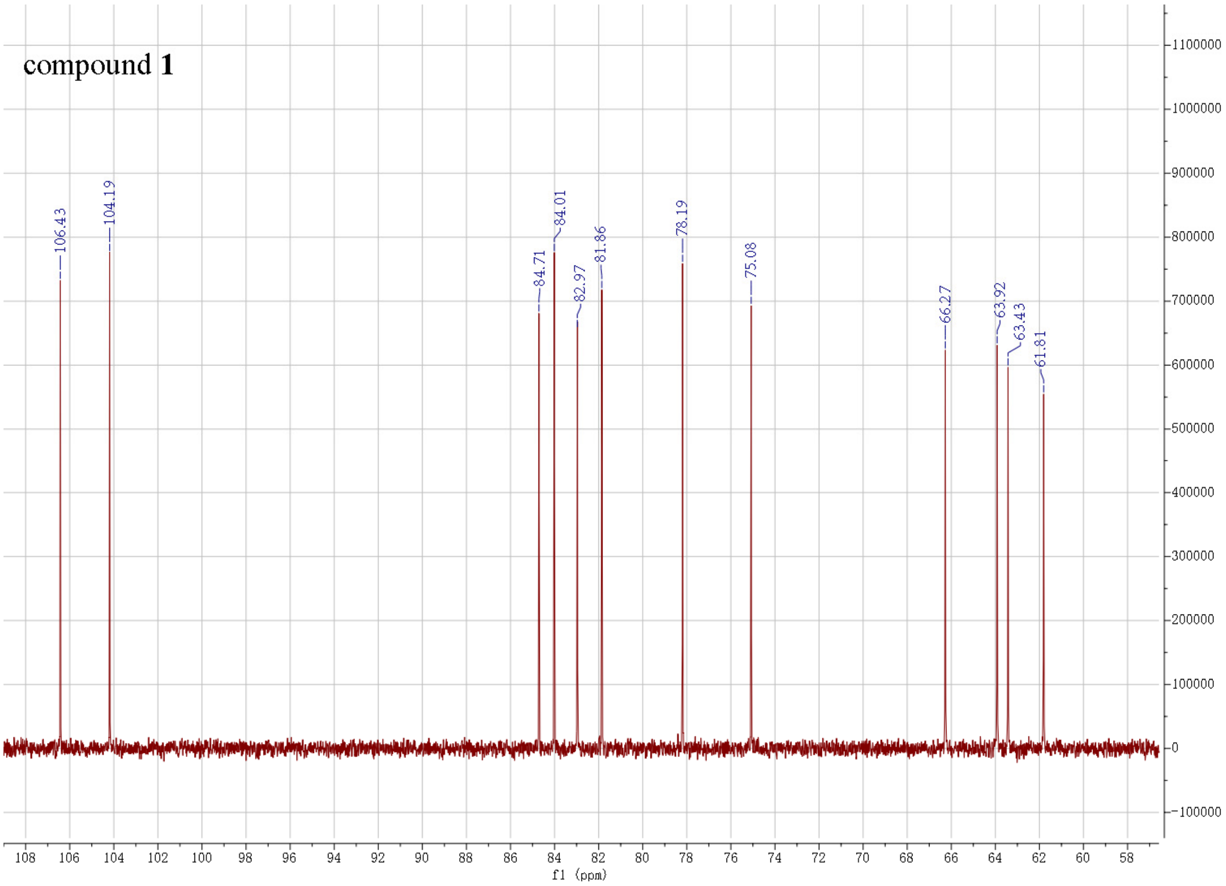
compound 1
106.43
104.19
84.71
84.01
82.97
81.86
78.19
75.08
66.27
63.92
63.43
61.81
f1 (ppm)

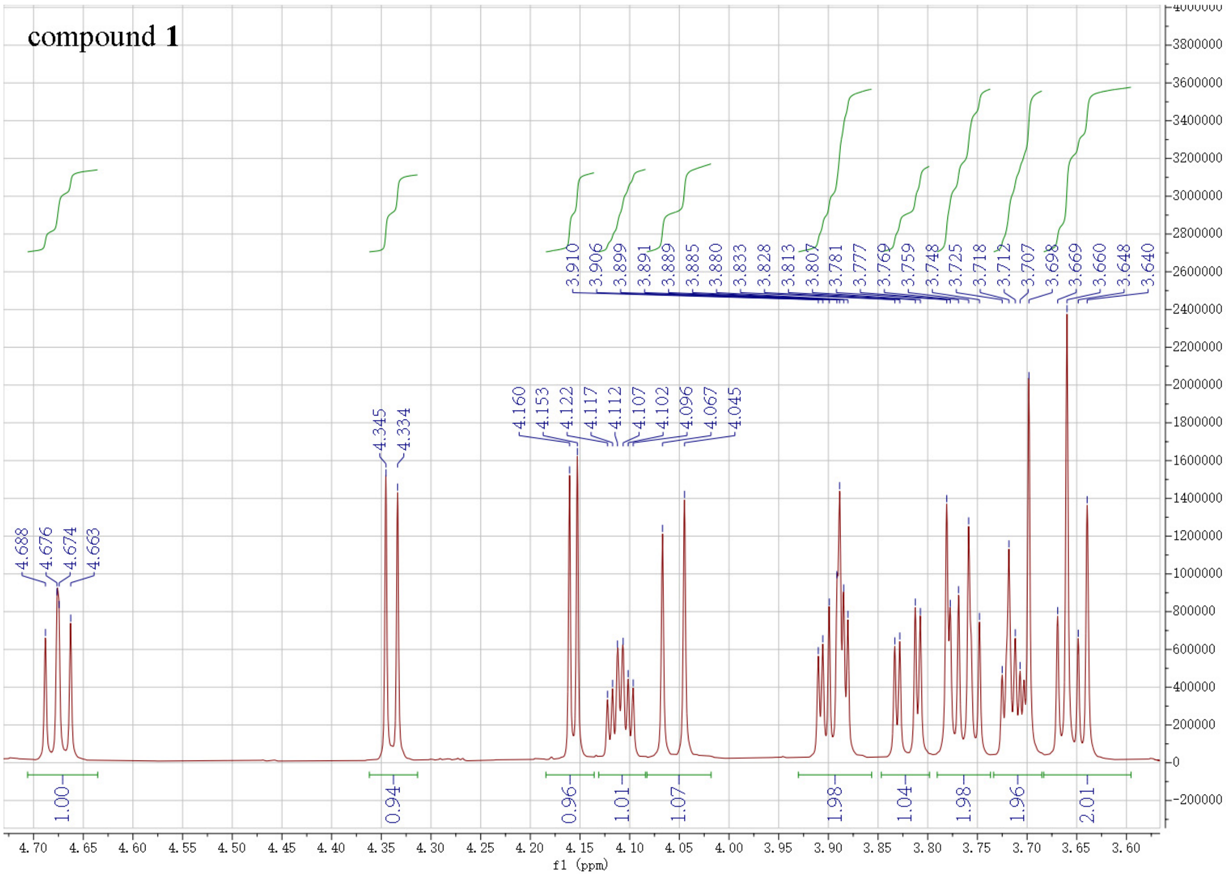
compound 1
4.688
4.676
4.674
4.663
4.345
4.334
4.160
4.153
4.122
4.117
4.112
4.107
4.102
4.096
4.067
4.045
3.910
3.906
3.899
3.891
3.889
3.885
3.880
3.833
3.828
3.813
3.807
3.781
3.777
3.769
3.759
3.748
3.725
3.718
3.712
3.707
3.698
3.669
3.660
3.648
3.640
1.00
0.94
0.96
1.01
1.07
1.98
1.04
1.98
1.96
2.01
f1 (ppm)

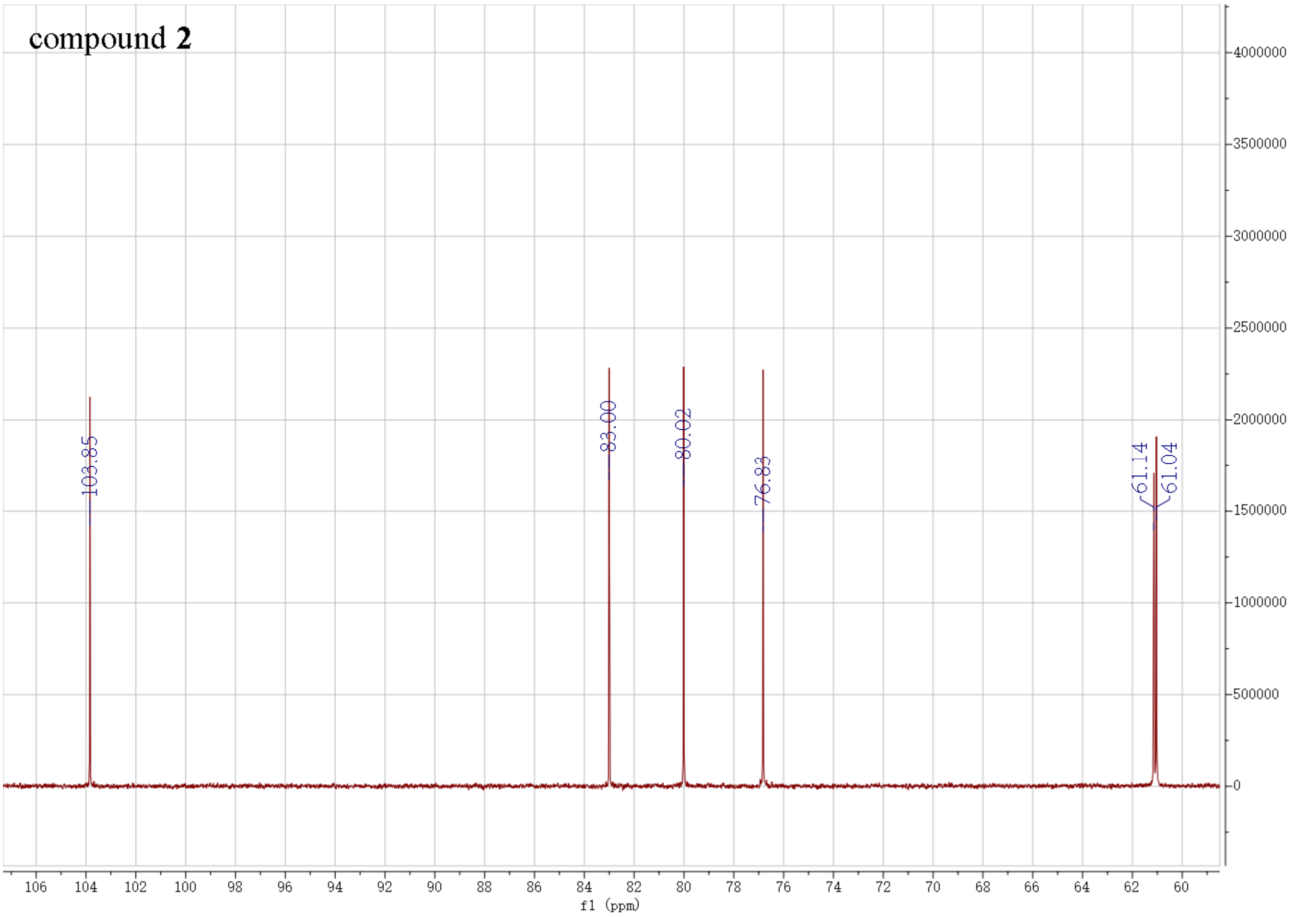
compound 2
103.85
83.00
80.02
76.83
61.14
61.04
f1 (ppm)

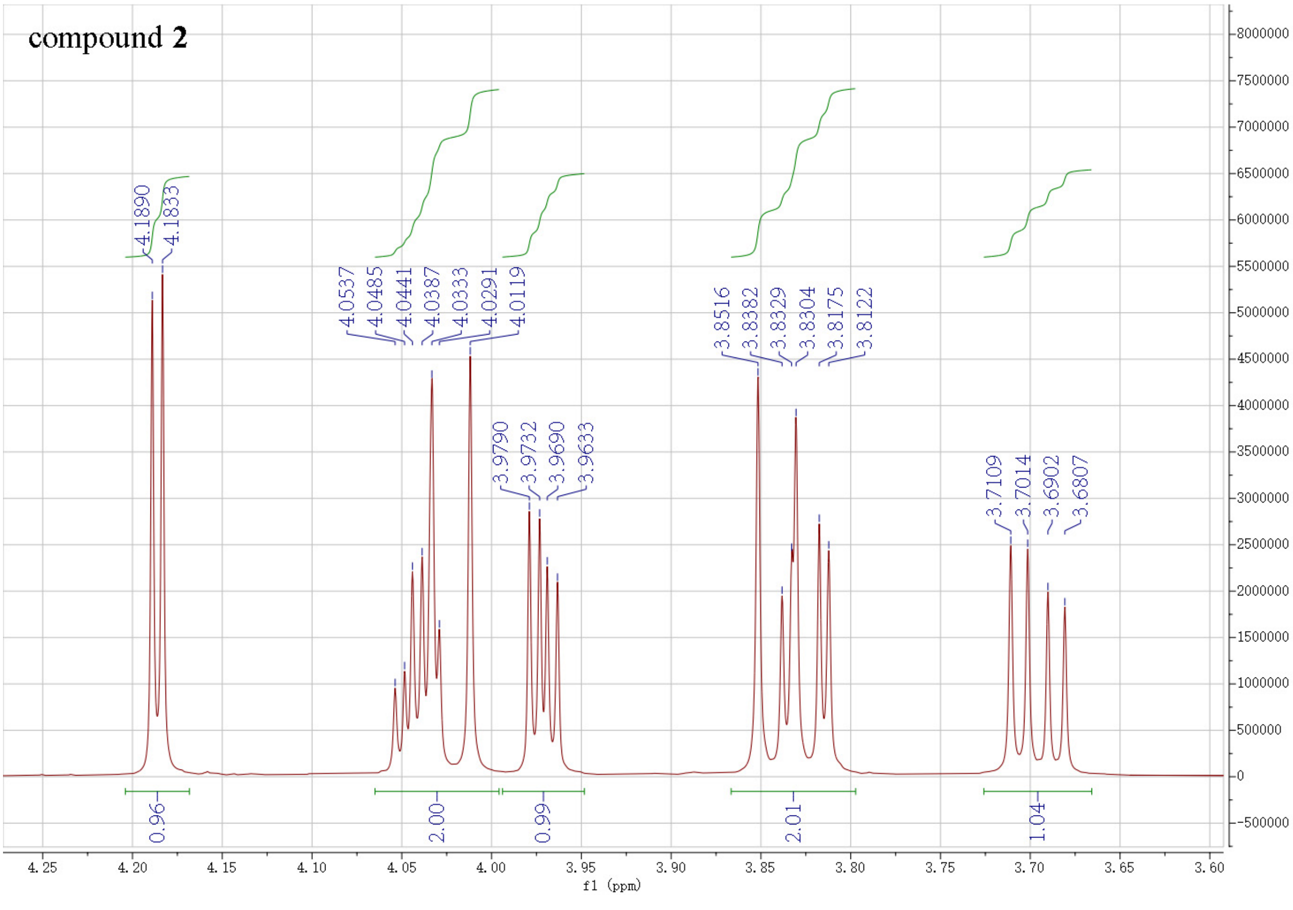
compound 2
4.1890
4.1833
4.0537
4.0485
4.0441
4.0387
4.0333
4.0291
4.0119
3.9790
3.9732
3.9690
3.9633
3.8516
3.8382
3.8329
3.8304
3.8175
3.8122
3.7109
3.7014
3.6902
3.6807
0.96
2.00
0.99
2.01
1.04
f1 (ppm)

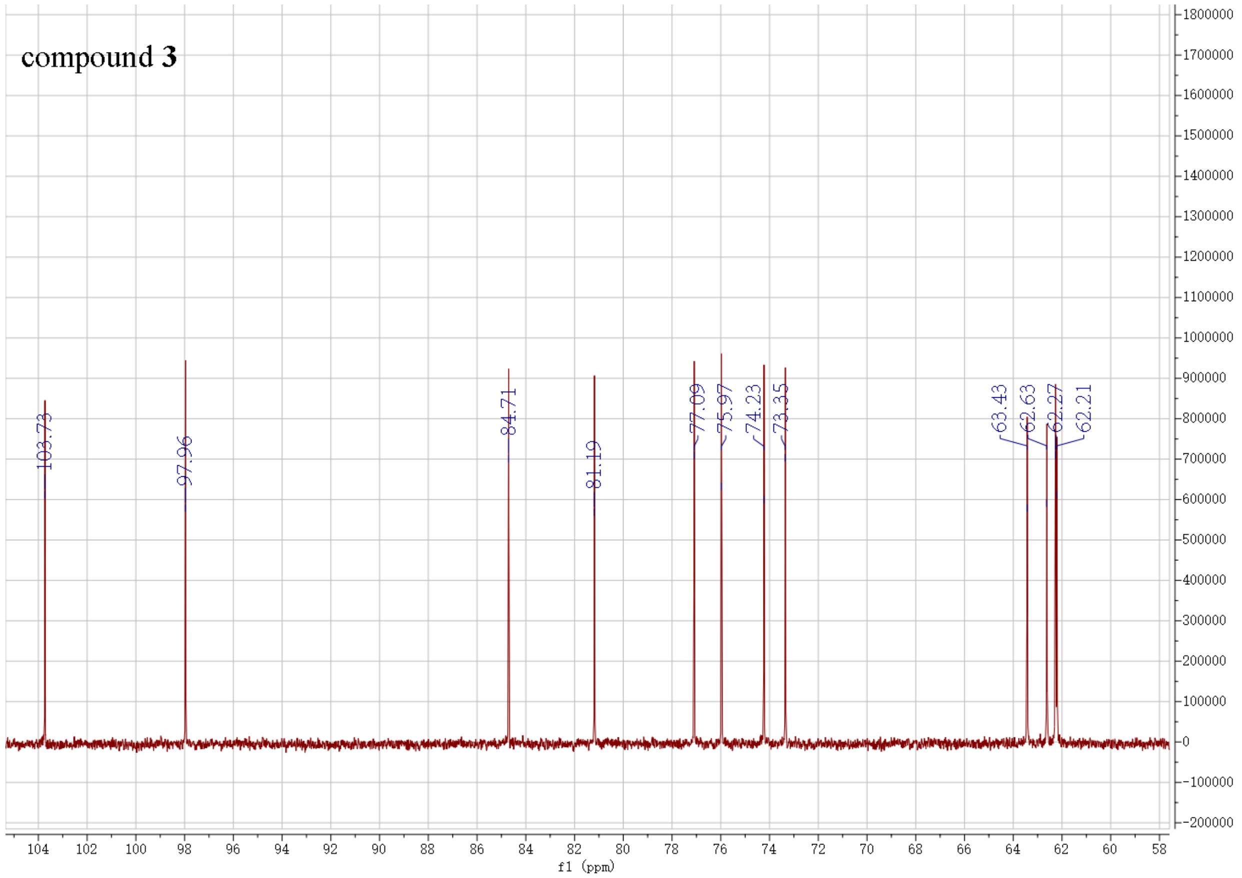
compound 3
103.73
97.96
84.71
81.19
77.09
75.97
74.23
73.35
63.43
62.63
62.27
62.21
f1 (ppm)

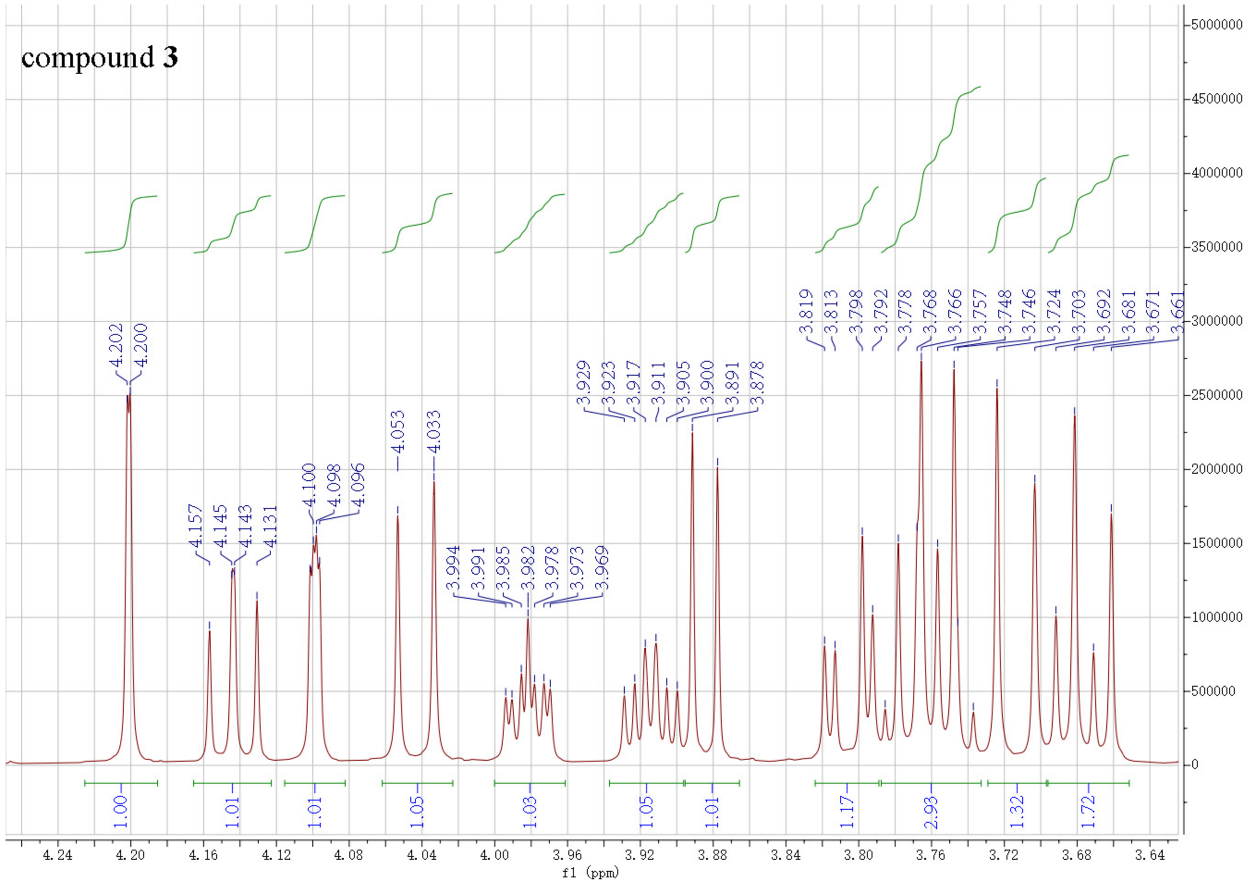
compound 3
4.202
4.200
4.157
4.145
4.143
4.131
4.100
4.098
4.096
4.053
4.033
3.994
3.991
3.985
3.982
3.978
3.973
3.969
3.929
3.923
3.917
3.911
3.905
3.900
3.891
3.878
3.819
3.813
3.798
3.792
3.778
3.768
3.766
3.757
3.748
3.746
3.724
3.703
3.692
3.681
3.671
3.661
1.00
1.01
1.01
1.05
1.03
1.05
1.01
1.17
2.93
1.32
1.72
f1 (ppm)

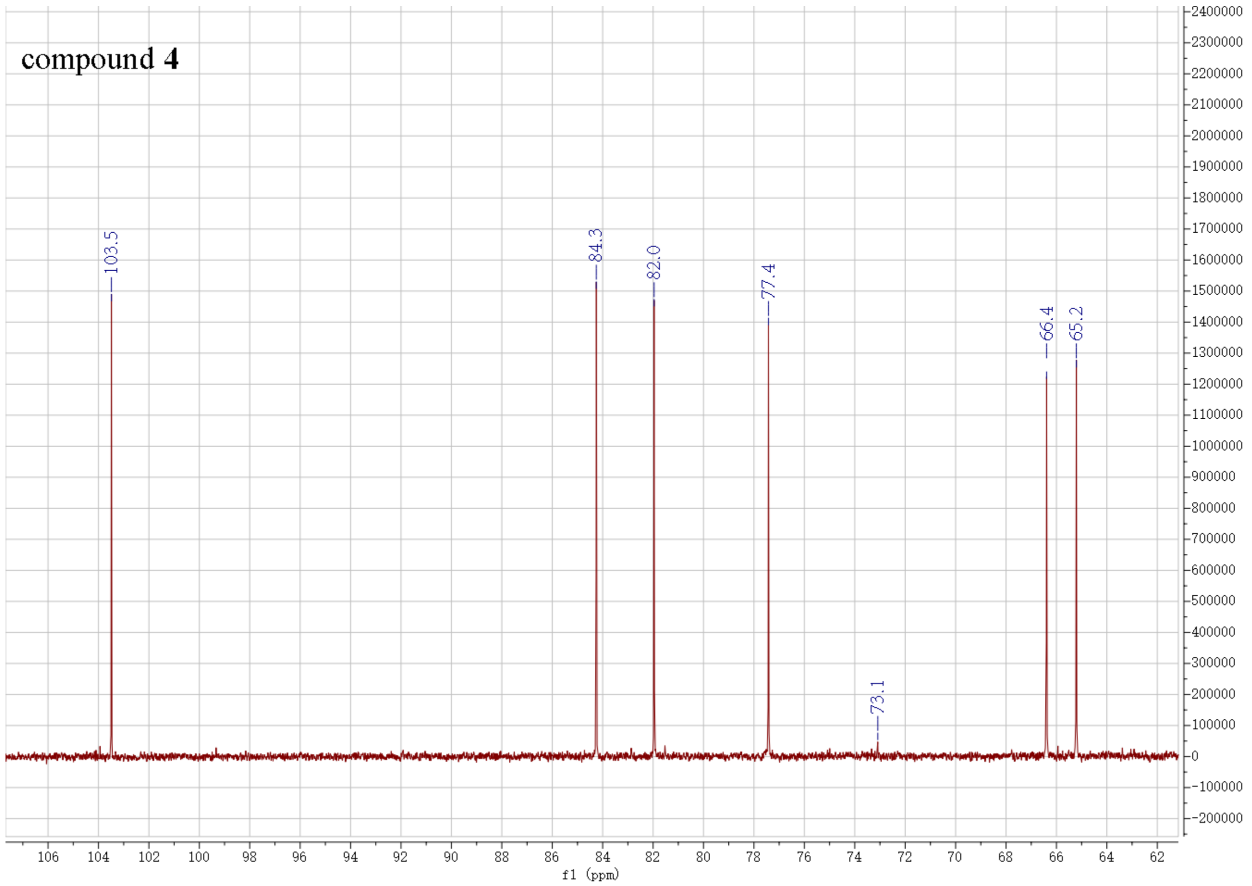
compound 4
103.5
84.3
82.0
77.4
73.1
66.4
65.2
f1 (ppm)

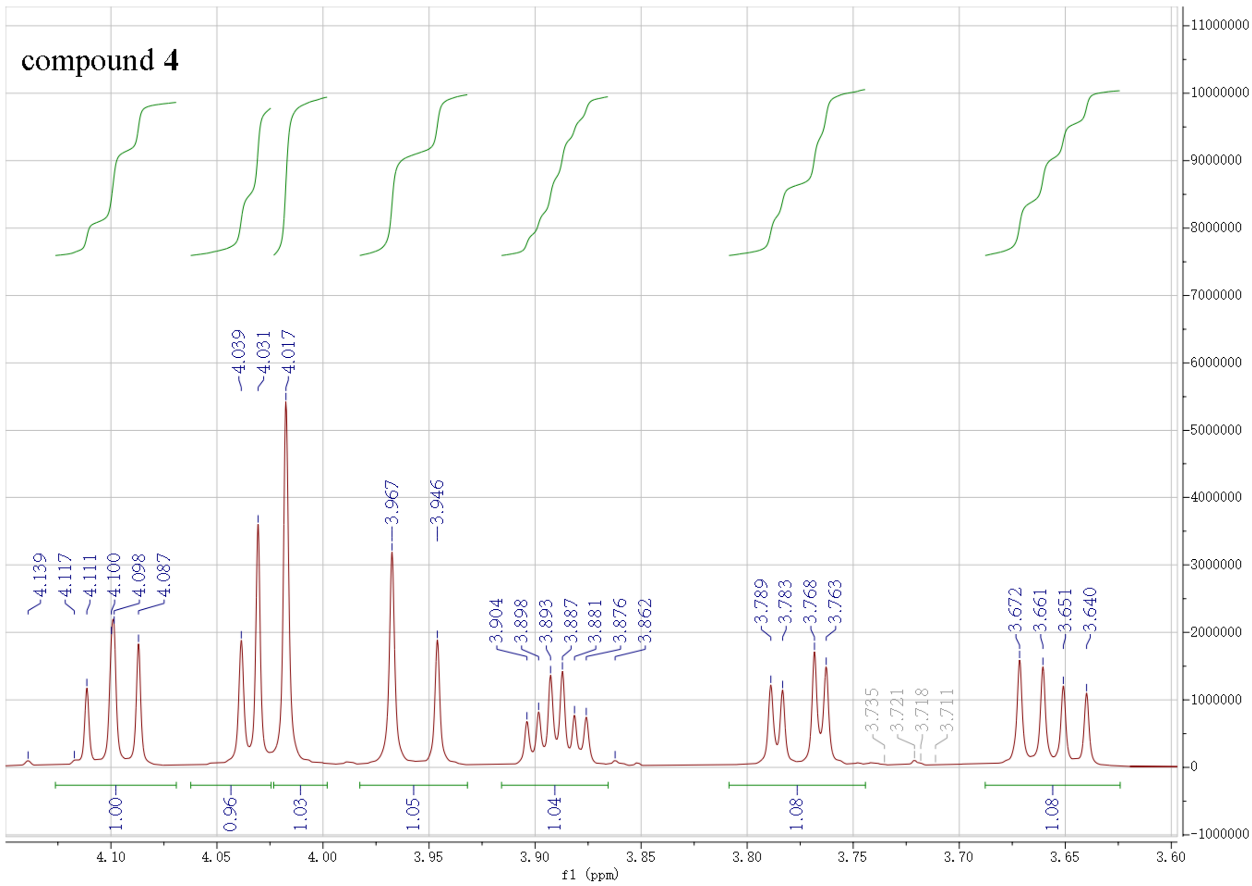
compound 4
4.139
4.117
4.111
4.100
4.098
4.087
4.039
4.031
4.017
3.967
3.946
3.904
3.898
3.893
3.887
3.881
3.876
3.862
3.789
3.783
3.768
3.763
3.735
3.721
3.718
3.711
3.672
3.661
3.651
3.640
1.00
0.96
1.03
1.05
1.04
1.08
1.08
f1 (ppm)

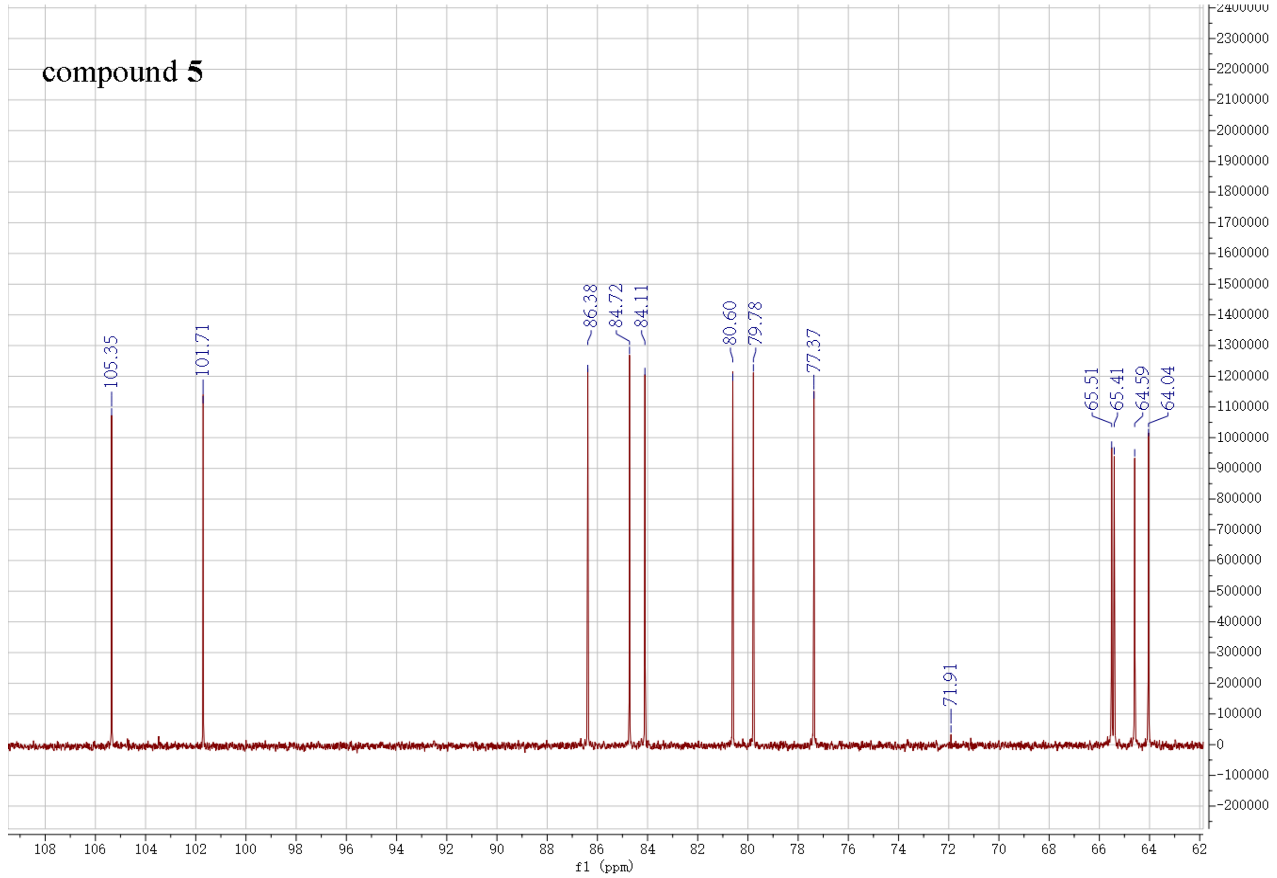
compound 5
105.35
101.71
86.38
84.72
84.11
80.60
79.78
77.37
71.91
65.51
65.41
64.59
64.04
f1 (ppm)

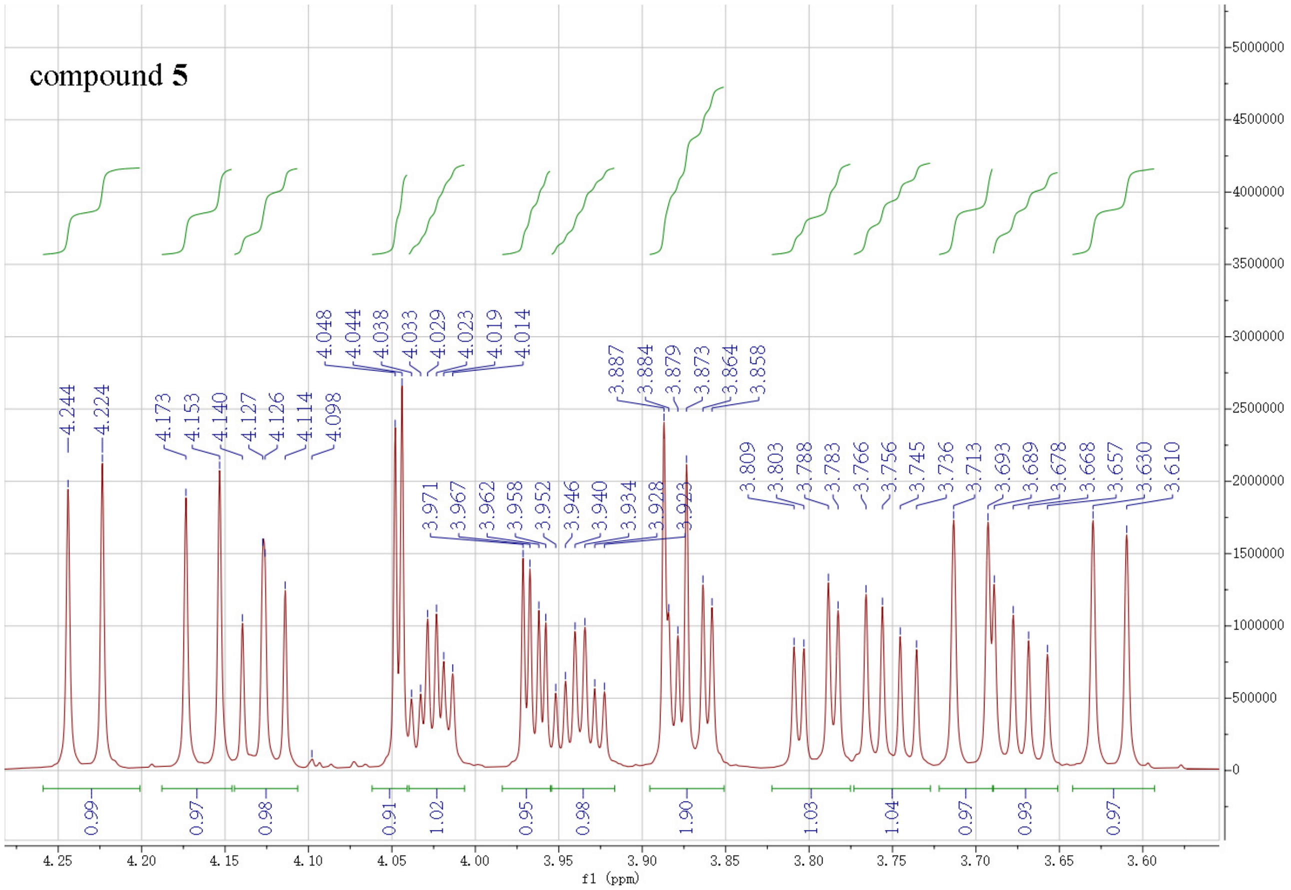
compound 5
4.244
4.224
4.173
4.153
4.140
4.127
4.126
4.114
4.098
4.048
4.044
4.038
4.033
4.029
4.023
4.019
4.014
3.971
3.967
3.962
3.958
3.952
3.946
3.940
3.934
3.928
3.923
3.887
3.884
3.879
3.873
3.864
3.858
3.809
3.803
3.788
3.783
3.766
3.756
3.745
3.736
3.713
3.693
3.689
3.678
3.668
3.657
3.630
3.610
0.99
0.97
0.98
0.91
1.02
0.95
0.98
1.90
1.03
1.04
0.97
0.93
0.97
f1 (ppm)

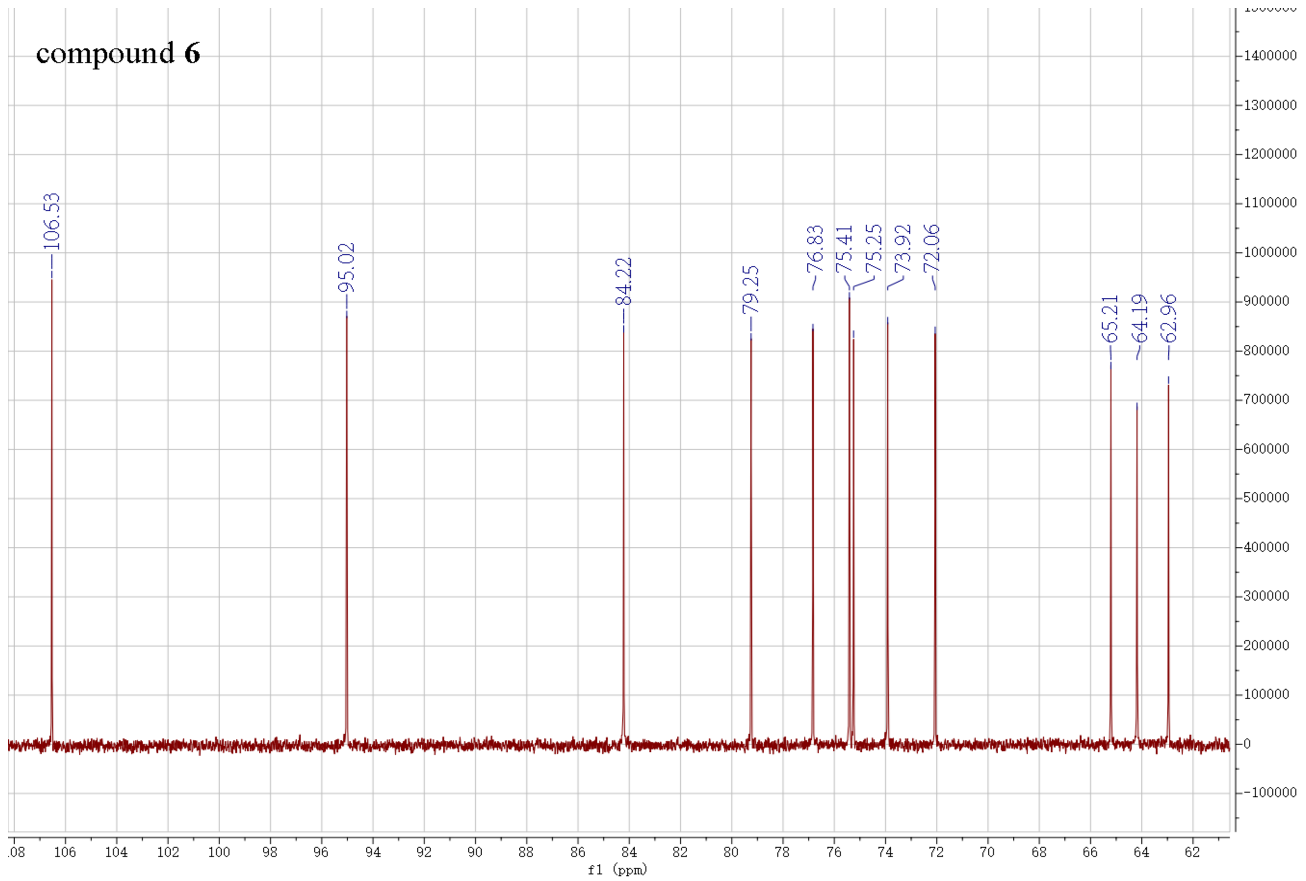
compound 6
106.53
95.02
84.22
79.25
76.83
75.41
75.25
73.92
72.06
65.21
64.19
62.96
f1 (ppm)

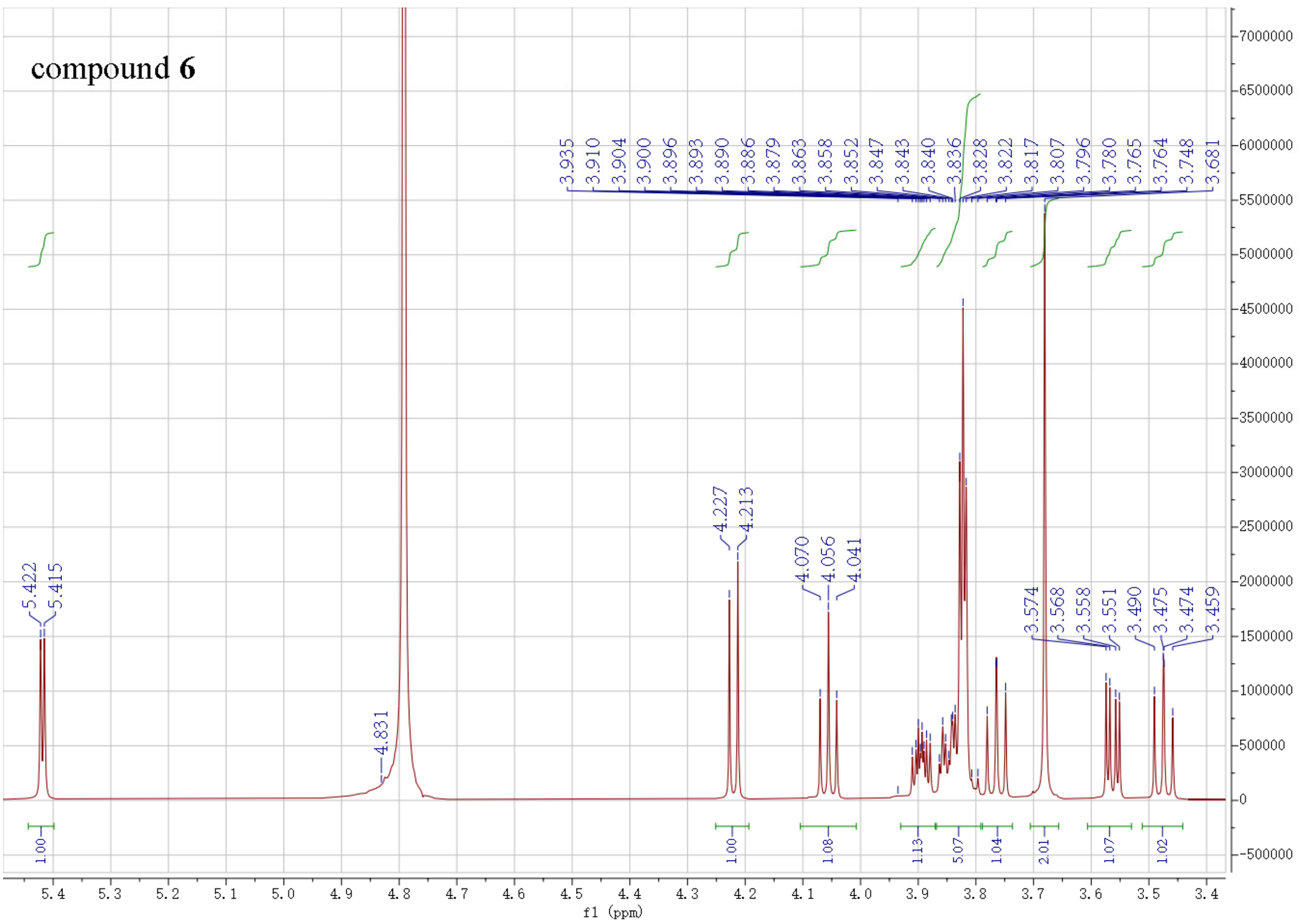
compound 6
3.935 3.910 3.904 3.900 3.896 3.893 3.890 3.886 3.879 3.863 3.858 3.852 3.847 3.843 3.840 3.836 3.828 3.822 3.817 3.807 3.796 3.780 3.765 3.764 3.748 3.681
5.422
5.415
4.831
4.227
4.213
4.070
4.056
4.041
3.574 3.568 3.558 3.551 3.490 3.475 3.474 3.459
1.00 1.00 1.08 1.13 5.07 1.04 2.01 1.07 1.02
f1 (ppm)

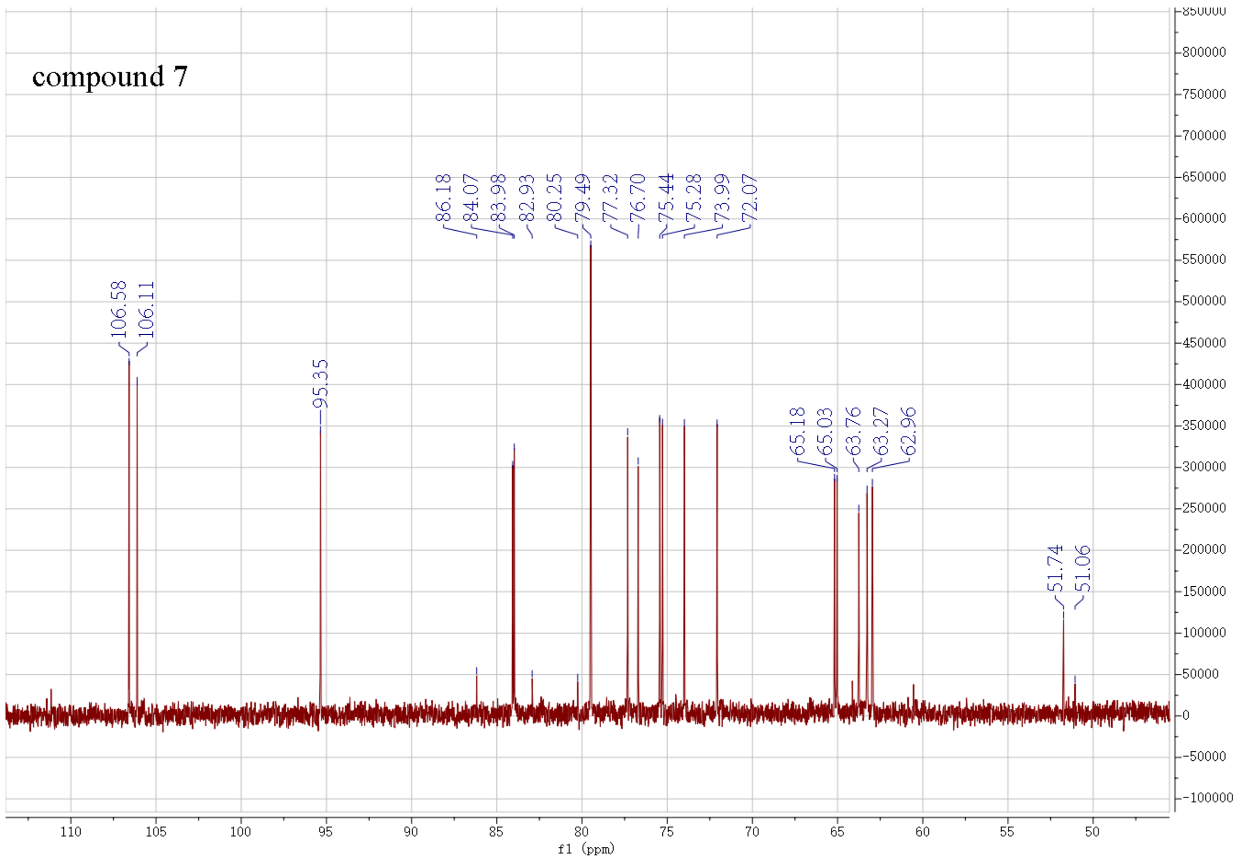

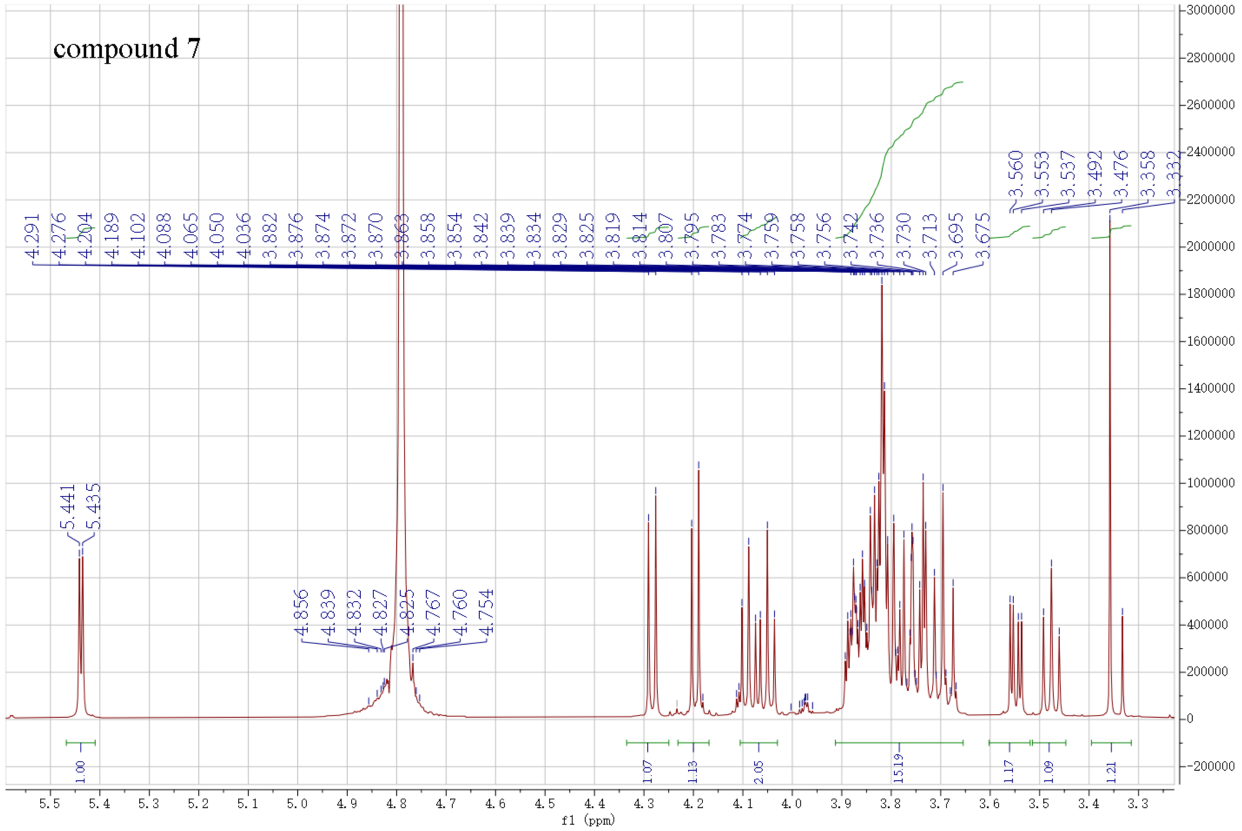

Figure S1. The 1H and 13C NMR spectra of compounds 1-7.

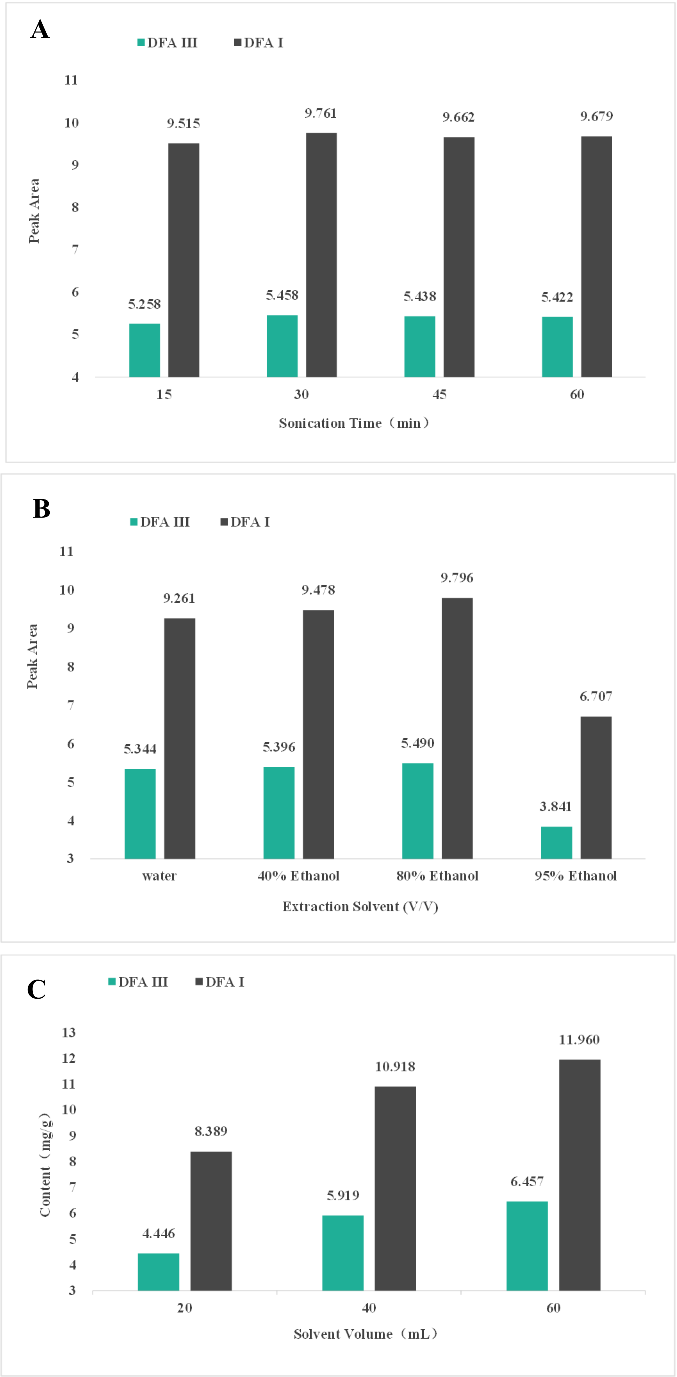

Figure S2. Extraction efficiency of two DFAs under different conditions; (A) Sonication time; (B) Extraction solvent. (C) Solvent volume.
